# Supplementary material for: Modelling Skylarks (Alauda arvensis) to Predict Impacts of Changes in Land Management and Policy: Development and Testing of an Agent-Based Model
Source: PLoS One. 2013 Jun 6;8(6):e65803. doi: 10.1371/journal.pone.0065803 (PMC3675089; doi:10.1371/journal.pone.0065803)
Supplement: Supporting Information S4 — The skylark ODdox as a zipped archive. (ZIP) [file pone.0065803.s004.zip › Skylark_ODdox/class_veg_element.html]

ALMaSS Skylark ODdox: VegElement Class Reference


ALMaSS Skylark ODdox
 2.0


- Main Page
- Related Pages
- Classes
- Files

- Class List
- Class Index
- Class Hierarchy
- Class Members

Public Member Functions |
Protected Member Functions |
Protected Attributes

VegElement Class Reference

`#include <elements.h>`

List of all members.

|  |  |
| --- | --- |
| Public Member Functions | |
| virtual void | DoDevelopment (void) |
| virtual void | ForceGrowthDevelopment (void) |
| virtual void | ForceGrowthInitialize (void) |
| virtual void | ForceGrowthTest (void) |
| virtual double | GetDeadBiomass (void) |
| virtual double | GetDigestability (void) |
| virtual double | GetGreenBiomass (void) |
| virtual double | GetInsectPop (void) |
| virtual double | GetLAGreen (void) |
| virtual double | GetLATotal (void) |
| virtual bool | GetSkScrapes (void) |
| virtual double | GetVegBiomass (void) |
| virtual double | GetVegCover (void) |
| virtual int | GetVegDensity (void) |
| virtual double | GetVegHeight (void) |
| virtual bool | GetVegPatchy (void) |
| virtual TTypesOfVegetation | GetVegType (void) |
| virtual double | GetWeedBiomass (void) |
| virtual void | Insecticide (double a\_fraction) |
| virtual void | InsectMortality (double a\_fraction) |
| virtual void | ReduceVeg (double a\_reduc) |
| virtual void | ReduceVeg\_Extended (double a\_reduc) |
| virtual void | ReduceWeedBiomass (double a\_fraction) |
| virtual void | SetCropData (double, double, double, TTypesOfVegetation, double, bool) |
| virtual void | SetCropDataAll (double, double, double, double, TTypesOfVegetation, double, double, bool, double, bool, double) |
| virtual void | SetGrowthPhase (int a\_phase) |
| virtual void | SetInsectPop (double insects) |
| virtual void | SetVegHeight (double a\_veg\_height, double a\_LAtotal, double a\_LAgreen, double a\_WeedBiomass) |
| virtual void | SetVegPatchy (bool p) |
| virtual void | SetVegType (TTypesOfVegetation a\_vege\_type, TTypesOfVegetation a\_weed\_type) |
| virtual void | StoreLAItotal () |
| virtual void | ToggleCattleGrazing (void) |
| virtual void | TogglePigGrazing (void) |
|  | VegElement (void) |
| virtual void | ZeroVeg (void) |
| virtual | ~VegElement (void) |
| Public Member Functions inherited from LE | |
| void | AddArea (double a\_area\_diff) |
| void | BumpRunNum (void) |
| double | GetArea (void) |
| LE \* | GetBorder (void) |
| bool | GetCattleGrazing (void) |
| virtual int | GetCentroidX () |
| virtual int | GetCentroidY () |
| int | GetCountryDesignation (void) |
| virtual double | GetDayDegrees (void) |
| virtual TTypesOfLandscapeElement | GetElementType (void) |
| int | GetFileType (void) |
| bool | GetHigh (void) |
| int | GetLastTreatment (int \*a\_index) |
| int | GetMapIndex (void) |
| bool | GetMapValid (void) |
| int | GetMaxX (void) |
| int | GetMaxY (void) |
| int | GetMConstants (int a) |
| int | GetMDates (int a, int b) |
| long | GetMgtLoopDetectCount (void) |
| long | GetMgtLoopDetectDate (void) |
| int | GetMinX (void) |
| int | GetMinY (void) |
| long | GetOldDays (void) |
| Farm \* | GetOwner (void) |
| int | GetOwnerFile (void) |
| int | GetOwnerIndex (void) |
| int | GetPesticideCell () |
| bool | GetPigGrazing (void) |
| bool | GetPoison (void) |
| int | GetPoly (void) |
| int | GetRotIndex (void) |
| long | GetRunNum (void) |
| LE\_Signal | GetSignal (void) |
| int | GetSoilType () |
| int | GetSubType (void) |
| virtual double | GetTrafficLoad (void) |
| int | GetUnsprayedMarginPolyRef (void) |
| int | GetValidX (void) |
| int | GetValidY (void) |
| int | GetVegAge () |
| int | GetVegStore (void) |
| bool | HasTramlines (void) |
| bool | IsRecentlyMown (void) |
| int | IsRecentlySprayed (void) |
|  | LE (void) |
| void | ResetTrace (void) |
| void | SetArea (double a\_area) |
| void | SetBorder (LE \*a\_border) |
| virtual void | SetCentroid (int x, int y) |
| void | SetCopyTreatment (int a\_treatment) |
| void | SetCountryDesignation (int a\_designation) |
| void | SetElementType (TTypesOfLandscapeElement a\_type) |
| void | SetFileType (int a\_file\_type) |
| void | SetHerbicideDelay (int a\_decaytime\_days) |
| void | SetHigh (bool a\_high) |
| void | SetLastTreatment (int a\_treatment) |
| void | SetMapIndex (int a\_map\_index) |
| void | SetMapValid (bool a\_valid) |
| void | SetMaxX (int x) |
| void | SetMaxY (int y) |
| void | SetMConstants (int a, int c) |
| void | SetMDates (int a, int b, int c) |
| void | SetMgtLoopDetectCount (long a\_num) |
| void | SetMgtLoopDetectDate (long a\_num) |
| void | SetMinX (int x) |
| void | SetMinY (int y) |
| void | SetMownDecay (int a\_decaytime\_days) |
| void | SetOldDays (long a\_days) |
| void | SetOwner (Farm \*a\_owner, int a\_owner\_num, int a\_owner\_index) |
| void | SetPesticideCell (int a\_cell) |
| void | SetPoison (bool a\_poison) |
| void | SetPoly (int a\_poly) |
| void | SetRotIndex (int a\_index) |
| void | SetSignal (LE\_Signal a\_signal) |
| void | SetSoilType (int a\_st) |
| void | SetSubType (int a\_subtype) |
| void | SetTramlinesDecay (int a\_decaytime\_days) |
| void | SetUnsprayedMarginPolyRef (int a\_unsprayedmargin) |
| void | SetValidXY (int a\_valid\_x, int a\_valid\_y) |
| virtual void | SetVegBiomass (int) |
| void | SetVegStore (int a\_veg) |
| void | Tick (void) |
| void | Trace (int a\_value) |
| void | ZeroVegAge () |
| virtual | ~LE (void) |

|  |  |
| --- | --- |
| Protected Member Functions | |
| void | ForceGrowthSpringTest (void) |
| void | RandomVegStartValues (double \*a\_LAtotal, double \*a\_LAgreen, double \*a\_veg\_height, double \*a\_weed\_biomass) |
| void | ReadBugPercentageFile (void) |
| virtual void | RecalculateBugsNStuff (void) |

|  |  |
| --- | --- |
| Protected Attributes | |
| int | m\_curve\_num |
| double | m\_dead\_biomass |
| double | m\_digestability |
| bool | m\_force\_growth |
| double | m\_force\_LAgreen |
| double | m\_force\_LAtotal |
| double | m\_force\_veg\_height |
| double | m\_force\_Weed |
| bool | m\_forced\_phase\_shift |
| double | m\_green\_biomass |
| FILE \* | m\_ifile |
| double | m\_insect\_pop |
| double | m\_LAgreen |
| double | m\_LAtotal |
| double | m\_newgrowthsum |
| int | m\_newoldgrowthindex |
| int | m\_nutrient\_status |
| double | m\_oldLAtotal |
| double | m\_oldnewgrowth [32] |
| double | m\_veg\_biomass |
| double | m\_veg\_cover |
| int | m\_veg\_density |
| double | m\_veg\_height |
| bool | m\_veg\_patchy |
| int | m\_veg\_phase |
| TTypesOfVegetation | m\_vege\_type |
| double | m\_weed\_biomass |
| int | m\_weed\_curve\_num |
| Protected Attributes inherited from LE | |
| double | m\_area |
| LE \* | m\_border |
| bool | m\_cattle\_grazing |
| int | m\_centroidx |
| int | m\_centroidy |
| int | m\_countrydesignation |
| int | m\_days\_since\_insecticide\_spray |
| double | m\_ddegs |
| int | m\_farmfunc\_tried\_to\_do |
| int | m\_file\_type |
| int | m\_herbicidedelay |
| bool | m\_high |
| bool | m\_is\_in\_map |
| unsigned int | m\_lastindex |
| vector< int > | m\_lasttreat |
| long | m\_management\_loop\_detect\_count |
| long | m\_management\_loop\_detect\_date |
| int | m\_map\_index |
| int | m\_maxx |
| int | m\_maxy |
| int | m\_minx |
| int | m\_miny |
| int | m\_mowndecay |
| long | m\_olddays |
| Farm \* | m\_owner |
| int | m\_owner\_file |
| int | m\_owner\_index |
| int | m\_PesticideGridCell |
| bool | m\_pig\_grazing |
| bool | m\_poison |
| int | m\_poly |
| int | m\_rot\_index |
| long | m\_running |
| LE\_Signal | m\_signal\_mask |
| int | m\_soiltype |
| int | m\_subtype |
| int | m\_tramlinesdecay |
| TTypesOfLandscapeElement | m\_type |
| int | m\_unsprayedmarginpolyref |
| int | m\_valid\_x |
| int | m\_valid\_y |
| int | m\_vegage |
| int | m\_vege\_danger\_store |
| double | m\_yddegs |
| int | MConsts [10] |
| int | MDates [2][25] |

|  |  |
| --- | --- |
| Additional Inherited Members | |
| Public Attributes inherited from LE | |
| bool | m\_repeat\_start |
| bool | m\_skylarkscrapes |
|  | For management testing of skylark scrapes. |
| int | m\_squares\_in\_map |
| int | m\_tried\_to\_do |
| long | m\_user [EL\_MAX\_USERSPACE] |
|  | Variable used to record the current grazing pressure by e.g. voles. |
| Static Protected Attributes inherited from LE | |
| static double | m\_largeroad\_load [] |
| static double | m\_monthly\_traffic [] |
| static double | m\_smallroad\_load [] |

---

## Constructor & Destructor Documentation

|  |  |  |  |  |  |
| --- | --- | --- | --- | --- | --- |
| VegElement::VegElement | ( | void |  | ) |  |

References EL\_BUG\_PERCENT\_A, EL\_BUG\_PERCENT\_B, EL\_BUG\_PERCENT\_C, EL\_BUG\_PERCENT\_D, EL\_BUG\_PERCENT\_Edges\_A, EL\_BUG\_PERCENT\_Edges\_B, EL\_BUG\_PERCENT\_Edges\_C, EL\_BUG\_PERCENT\_G\_A, EL\_BUG\_PERCENT\_G\_B, EL\_BUG\_PERCENT\_G\_C, EL\_BUG\_PERCENT\_SB\_A, EL\_BUG\_PERCENT\_SB\_B, EL\_BUG\_PERCENT\_SB\_C, EL\_BUG\_PERCENT\_WR\_A, EL\_BUG\_PERCENT\_WR\_B, EL\_BUG\_PERCENT\_WR\_C, EL\_BUG\_PERCENT\_WRy\_A, EL\_BUG\_PERCENT\_WRy\_B, EL\_BUG\_PERCENT\_WRy\_C, EL\_BUG\_PERCENT\_WW\_A, EL\_BUG\_PERCENT\_WW\_B, EL\_BUG\_PERCENT\_WW\_C, g\_biomass\_scale, g\_bug\_percent\_a, g\_bug\_percent\_b, g\_bug\_percent\_c, g\_bug\_percent\_d, g\_crops, g\_weed\_percent, janfirst, l\_el\_read\_bug\_percentage\_file(), m\_curve\_num, LE::m\_ddegs, m\_digestability, m\_force\_growth, m\_forced\_phase\_shift, m\_insect\_pop, m\_LAgreen, m\_LAtotal, m\_newgrowthsum, m\_newoldgrowthindex, m\_nutrient\_status, m\_oldnewgrowth, m\_veg\_biomass, m\_veg\_cover, m\_veg\_height, m\_vege\_type, m\_weed\_biomass, m\_weed\_curve\_num, LE::m\_yddegs, SetGrowthPhase(), SetVegPatchy(), tov\_AgroChemIndustryCereal, tov\_Carrots, tov\_CloverGrassGrazed1, tov\_CloverGrassGrazed2, tov\_FieldPeas, tov\_FieldPeasStrigling, tov\_FodderBeet, tov\_FodderGrass, tov\_Maize, tov\_MaizeSilage, tov\_MaizeStrigling, tov\_NaturalGrass, tov\_NoGrowth, tov\_None, tov\_Oats, tov\_OBarleyPeaCloverGrass, tov\_OCarrots, tov\_OCloverGrassGrazed1, tov\_OCloverGrassGrazed2, tov\_OCloverGrassSilage1, tov\_OFieldPeas, tov\_OFieldPeasSilage, tov\_OFirstYearDanger, tov\_OGrazingPigs, tov\_OMaizeSilage, tov\_OOats, tov\_OPermanentGrassGrazed, tov\_OPotatoes, tov\_OSBarleySilage, tov\_OSeedGrass1, tov\_OSeedGrass2, tov\_OSetaside, tov\_OSpringBarley, tov\_OSpringBarleyClover, tov\_OSpringBarleyExt, tov\_OSpringBarleyGrass, tov\_OSpringBarleyPigs, tov\_OTriticale, tov\_OWinterBarley, tov\_OWinterBarleyExt, tov\_OWinterRape, tov\_OWinterRye, tov\_OWinterWheatUndersown, tov\_OWinterWheatUndersownExt, tov\_PermanentGrassGrazed, tov\_PermanentGrassLowYield, tov\_PermanentGrassTussocky, tov\_PermanentSetaside, tov\_Potatoes, tov\_PotatoesIndustry, tov\_SeedGrass1, tov\_SeedGrass2, tov\_Setaside, tov\_SpringBarley, tov\_SpringBarleyCloverGrass, tov\_SpringBarleyCloverGrassStrigling, tov\_SpringBarleyGrass, tov\_SpringBarleyPeaCloverGrassStrigling, tov\_SpringBarleyPTreatment, tov\_SpringBarleySeed, tov\_SpringBarleySilage, tov\_SpringBarleySKManagement, tov\_SpringBarleyStrigling, tov\_SpringBarleyStriglingCulm, tov\_SpringBarleyStriglingSingle, tov\_SpringRape, tov\_SpringWheat, tov\_Triticale, tov\_WinterBarley, tov\_WinterBarleyStrigling, tov\_WinterRape, tov\_WinterRapeStrigling, tov\_WinterRye, tov\_WinterRyeStrigling, tov\_WinterWheat, tov\_WinterWheatShort, tov\_WinterWheatStrigling, tov\_WinterWheatStriglingCulm, tov\_WinterWheatStriglingSingle, tov\_WWheatPControl, tov\_WWheatPToxicControl, tov\_WWheatPTreatment, tov\_YoungForest, CfgBool::value(), and CropData::VegTypeToCurveNum().

: LE() {

SetVegPatchy( false );

m\_nutrient\_status = 0; // default

m\_veg\_biomass = 0.0;

m\_weed\_biomass = 0.0;

m\_veg\_height = 0.0;

m\_veg\_cover = 0.0;

m\_insect\_pop = 1.0;

m\_vege\_type = tov\_NoGrowth;

m\_curve\_num = g\_crops->VegTypeToCurveNum( m\_vege\_type );

m\_weed\_curve\_num = 99; // 99 used as zero growth curve

m\_yddegs = 0.0;

m\_ddegs = 0.0;

m\_LAgreen = 0.0;

m\_LAtotal = 0.0;

m\_digestability=1.0;

for (int i=0; i<16; i++) m\_oldnewgrowth[i]=0.5;

m\_newoldgrowthindex=0;

m\_newgrowthsum=8.0;

m\_forced\_phase\_shift = false;

m\_force\_growth = false;

SetGrowthPhase( janfirst );

g\_biomass\_scale[ tov\_Carrots ] = 0.7857;

g\_biomass\_scale[ tov\_CloverGrassGrazed1 ] = 0.7857;

g\_biomass\_scale[ tov\_CloverGrassGrazed2 ] = 0.7857;

g\_biomass\_scale[ tov\_FodderGrass ] = 0.7857;

g\_biomass\_scale[ tov\_FieldPeas ] = 0.857;

g\_biomass\_scale[ tov\_FodderBeet ] = 0.857;

g\_biomass\_scale[ tov\_Maize ] = 1.00;

g\_biomass\_scale[ tov\_MaizeSilage ] = 1.00;

g\_biomass\_scale[ tov\_OMaizeSilage ] = 1.00;

g\_biomass\_scale[ tov\_NaturalGrass ] = 0.567; //0.567 is scaled for actual yield

g\_biomass\_scale[ tov\_NoGrowth ] = 0.0;

g\_biomass\_scale[ tov\_None ] = 0.0;

g\_biomass\_scale[ tov\_Oats ] = 0.857;

g\_biomass\_scale[ tov\_OBarleyPeaCloverGrass ] = 0.857;

g\_biomass\_scale[ tov\_OSBarleySilage ] = 0.857 \* 0.8;

g\_biomass\_scale[ tov\_OCarrots ] = 0.7857 \* 0.8;

g\_biomass\_scale[ tov\_OCloverGrassGrazed1 ] = 0.7857;

g\_biomass\_scale[ tov\_OCloverGrassGrazed2 ] = 0.7857;

g\_biomass\_scale[ tov\_OCloverGrassSilage1 ] = 0.7857;

g\_biomass\_scale[ tov\_OFieldPeas ] = 0.857 \* 0.8;

g\_biomass\_scale[ tov\_OFieldPeasSilage ] = 0.857 \* 0.8;

g\_biomass\_scale[ tov\_OFirstYearDanger ] = 0.0;

g\_biomass\_scale[ tov\_OGrazingPigs ] = 0.7857 \* 0.8;

g\_biomass\_scale[ tov\_OOats ] = 0.857 \* 0.8;

g\_biomass\_scale[ tov\_OPermanentGrassGrazed ] = 0.7857;

g\_biomass\_scale[ tov\_OPotatoes ] = 0.857 \* 0.8;

g\_biomass\_scale[ tov\_OSeedGrass1 ] = 0.7857;

g\_biomass\_scale[ tov\_OSeedGrass2 ] = 0.7857;

g\_biomass\_scale[ tov\_OSetaside ] = 0.7857;

g\_biomass\_scale[ tov\_OSpringBarley ] = 0.857 \* 0.8;

g\_biomass\_scale[ tov\_OSpringBarleyExt ] = 0.857 \* 0.8 \* 0.8;

g\_biomass\_scale[ tov\_OSpringBarleyClover ] = 0.857 \* 0.8;

g\_biomass\_scale[ tov\_OSpringBarleyGrass ] = 0.857 \* 0.8;

g\_biomass\_scale[ tov\_OSpringBarleyPigs ] = 0.857 \* 0.8;

g\_biomass\_scale[ tov\_OTriticale ] = 1.00 \* 0.8;

g\_biomass\_scale[ tov\_OWinterBarley ] = 0.857 \* 0.8;

g\_biomass\_scale[ tov\_OWinterBarleyExt ] = 0.857 \* 0.8 \* 0.8;

g\_biomass\_scale[ tov\_OWinterRape ] = 1.071 \* 0.8;

g\_biomass\_scale[ tov\_OWinterRye ] = 0.857 \* 0.8;

g\_biomass\_scale[ tov\_OWinterWheatUndersown ] = 1.00 \* 0.8;

g\_biomass\_scale[ tov\_OWinterWheatUndersownExt ] = 1.00 \* 0.8 \* 0.8;

g\_biomass\_scale[ tov\_PermanentGrassGrazed ] = 0.7857;

g\_biomass\_scale[ tov\_PermanentGrassLowYield ] = 0.7857;

g\_biomass\_scale[ tov\_PermanentGrassTussocky ] = 0.7857;

g\_biomass\_scale[ tov\_PermanentSetaside ] = 0.7857;

g\_biomass\_scale[ tov\_Potatoes ] = 0.857;

g\_biomass\_scale[ tov\_PotatoesIndustry ] = 0.857;

g\_biomass\_scale[ tov\_SeedGrass1 ] = 0.7857;

g\_biomass\_scale[ tov\_SeedGrass2 ] = 0.7857;

g\_biomass\_scale[ tov\_Setaside ] = 0.7857;

g\_biomass\_scale[ tov\_SpringBarley ] = 0.857;

g\_biomass\_scale[ tov\_SpringBarleyPTreatment ] = 0.857;

g\_biomass\_scale[ tov\_SpringBarleySKManagement ] = 0.857;

g\_biomass\_scale[ tov\_SpringBarleyCloverGrass ] = 0.857;

g\_biomass\_scale[ tov\_SpringBarleyGrass ] = 0.857;

g\_biomass\_scale[ tov\_SpringBarleySeed ] = 0.857;

g\_biomass\_scale[ tov\_SpringBarleySilage ] = 0.857;

g\_biomass\_scale[ tov\_SpringRape ] = 1.071;

g\_biomass\_scale[ tov\_SpringWheat ] = 1.00;

g\_biomass\_scale[ tov\_Triticale ] = 1.00;

g\_biomass\_scale[ tov\_WinterBarley ] = 0.857;

g\_biomass\_scale[ tov\_WinterRape ] = 1.071;

g\_biomass\_scale[ tov\_WinterRye ] = 0.857;

g\_biomass\_scale[ tov\_WinterWheat ] = 1.00;

g\_biomass\_scale[ tov\_WinterWheatShort ] = 1.00;

g\_biomass\_scale[ tov\_AgroChemIndustryCereal ] = 1.00;

g\_biomass\_scale[ tov\_WWheatPControl ] = 1.00;

g\_biomass\_scale[ tov\_WWheatPToxicControl ] = 1.00;

g\_biomass\_scale[ tov\_WWheatPTreatment ] = 1.00;

g\_biomass\_scale[ tov\_WinterWheatStrigling ] = 1.00;

g\_biomass\_scale[ tov\_WinterWheatStriglingCulm ] = 1.00;

g\_biomass\_scale[ tov\_WinterWheatStriglingSingle ] = 1.00;

g\_biomass\_scale[ tov\_SpringBarleyCloverGrassStrigling ] = 0.857;

g\_biomass\_scale[ tov\_SpringBarleyStrigling ] = 0.857;

g\_biomass\_scale[ tov\_SpringBarleyStriglingSingle ] = 0.857;

g\_biomass\_scale[ tov\_SpringBarleyStriglingCulm ] = 0.857;

g\_biomass\_scale[ tov\_MaizeStrigling ] = 0.857;

g\_biomass\_scale[ tov\_WinterRapeStrigling ] = 1.071;

g\_biomass\_scale[ tov\_WinterRyeStrigling ] = 0.857;

g\_biomass\_scale[ tov\_WinterBarleyStrigling ] = 0.857;

g\_biomass\_scale[ tov\_FieldPeasStrigling ] = 0.857;

g\_biomass\_scale[ tov\_SpringBarleyPeaCloverGrassStrigling ] = 0.857;

g\_biomass\_scale[ tov\_YoungForest ] = 0.7857 \* 0.67;

if ( l\_el\_read\_bug\_percentage\_file.value() ) {

//ReadBugPercentageFile();

return;

}

g\_weed\_percent[ tov\_Carrots ] = 0.1;

g\_weed\_percent[ tov\_Maize ] = 0.05;

g\_weed\_percent[ tov\_MaizeSilage ] = 0.05;

g\_weed\_percent[ tov\_OMaizeSilage ] = 0.05;

g\_weed\_percent[ tov\_OCarrots ] = 0.1;

g\_weed\_percent[ tov\_Potatoes ] = 0.1;

g\_weed\_percent[ tov\_OPotatoes ] = 0.1;

g\_weed\_percent[ tov\_FodderGrass] = 0.1;

g\_weed\_percent[ tov\_CloverGrassGrazed1 ] = 0.1;

g\_weed\_percent[ tov\_CloverGrassGrazed2 ] = 0.1;

g\_weed\_percent[ tov\_OCloverGrassGrazed1 ] = 0.1;

g\_weed\_percent[ tov\_OCloverGrassGrazed2 ] = 0.1;

g\_weed\_percent[ tov\_SpringBarley ] = 0.1;

g\_weed\_percent[ tov\_SpringBarleyPTreatment ] = 0.1;

g\_weed\_percent[ tov\_SpringBarleySKManagement ] = 0.1;

g\_weed\_percent[ tov\_WinterWheat ] = 0.1;

g\_weed\_percent[ tov\_WinterWheatShort ] = 0.1;

g\_weed\_percent[ tov\_SpringBarleySilage ] = 0.1;

g\_weed\_percent[ tov\_SpringBarleySeed ] = 0.1;

g\_weed\_percent[ tov\_OGrazingPigs ] = 0.1;

g\_weed\_percent[ tov\_OCloverGrassSilage1 ] = 0.1;

g\_weed\_percent[ tov\_SpringBarleyCloverGrass ] = 0.1;

g\_weed\_percent[ tov\_OSpringBarleyPigs ] = 0.1;

g\_weed\_percent[ tov\_OBarleyPeaCloverGrass ] = 0.1;

g\_weed\_percent[ tov\_OSpringBarley ] = 0.1;

g\_weed\_percent[ tov\_OSpringBarleyExt ] = 0.1;

g\_weed\_percent[ tov\_OSBarleySilage ] = 0.1;

g\_weed\_percent[ tov\_OWinterWheatUndersown ] = 0.1;

g\_weed\_percent[ tov\_OWinterWheatUndersownExt ] = 0.1;

g\_weed\_percent[ tov\_WinterRape ] = 0.05;

g\_weed\_percent[ tov\_OWinterRape ] = 0.05;

g\_weed\_percent[ tov\_OWinterRye ] = 0.1;

g\_weed\_percent[ tov\_OWinterBarley ] = 0.1;

g\_weed\_percent[ tov\_OWinterBarleyExt ] = 0.1;

g\_weed\_percent[ tov\_WinterBarley ] = 0.1;

g\_weed\_percent[ tov\_WinterRye ] = 0.1;

g\_weed\_percent[ tov\_OFieldPeas ] = 0.1;

g\_weed\_percent[ tov\_OFieldPeasSilage ] = 0.1;

g\_weed\_percent[ tov\_OOats ] = 0.1;

g\_weed\_percent[ tov\_Oats ] = 0.1;

g\_weed\_percent[ tov\_FieldPeas ] = 0.1;

g\_weed\_percent[ tov\_SeedGrass1 ] = 0.1;

g\_weed\_percent[ tov\_SeedGrass2 ] = 0.1;

g\_weed\_percent[ tov\_OPermanentGrassGrazed ] = 0.1;

g\_weed\_percent[ tov\_Setaside ] = 0.1;

g\_weed\_percent[ tov\_OSetaside ] = 0.1;

g\_weed\_percent[ tov\_PermanentSetaside ] = 0.1;

g\_weed\_percent[ tov\_PermanentGrassLowYield ] = 0.1;

g\_weed\_percent[ tov\_PermanentGrassGrazed ] = 0.1;

g\_weed\_percent[ tov\_PermanentGrassTussocky ] = 0.1;

g\_weed\_percent[ tov\_FodderBeet ] = 0.1;

g\_weed\_percent[ tov\_NaturalGrass ] = 0.1;

g\_weed\_percent[ tov\_None ] = 0.1;

g\_weed\_percent[ tov\_NoGrowth ] = 0.1;

g\_weed\_percent[ tov\_OFirstYearDanger ] = 0.1;

g\_weed\_percent[ tov\_Triticale ] = 0.1;

g\_weed\_percent[ tov\_WWheatPControl ] = 0.1;

g\_weed\_percent[ tov\_WWheatPToxicControl ] = 0.1;

g\_weed\_percent[ tov\_WWheatPTreatment ] = 0.1;

g\_weed\_percent[ tov\_AgroChemIndustryCereal ] = 0.1;

g\_weed\_percent[ tov\_WinterWheatStrigling ] = 0.1;

g\_weed\_percent[ tov\_WinterWheatStriglingSingle ] = 0.1;

g\_weed\_percent[ tov\_WinterWheatStriglingCulm ] = 0.1;

g\_weed\_percent[ tov\_SpringBarleyCloverGrassStrigling ] = 0.1;

g\_weed\_percent[ tov\_SpringBarleyStrigling ] = 0.1;

g\_weed\_percent[ tov\_SpringBarleyStriglingSingle ] = 0.1;

g\_weed\_percent[ tov\_SpringBarleyStriglingCulm ] = 0.1;

g\_weed\_percent[ tov\_MaizeStrigling ] = 0.1;

g\_weed\_percent[ tov\_WinterRapeStrigling ] = 0.1;

g\_weed\_percent[ tov\_WinterRyeStrigling ] = 0.1;

g\_weed\_percent[ tov\_WinterBarleyStrigling ] = 0.1;

g\_weed\_percent[ tov\_FieldPeasStrigling ] = 0.1;

g\_weed\_percent[ tov\_SpringBarleyPeaCloverGrassStrigling ] = 0.1;

g\_weed\_percent[ tov\_YoungForest ] = 0.1;

g\_bug\_percent\_a[ tov\_Carrots ] = EL\_BUG\_PERCENT\_A;

g\_bug\_percent\_a[ tov\_MaizeSilage ] = EL\_BUG\_PERCENT\_A;

g\_bug\_percent\_a[ tov\_OMaizeSilage ] = EL\_BUG\_PERCENT\_A;

g\_bug\_percent\_a[ tov\_Maize ] = EL\_BUG\_PERCENT\_A;

g\_bug\_percent\_a[ tov\_OCarrots ] = EL\_BUG\_PERCENT\_A;

g\_bug\_percent\_a[ tov\_Potatoes ] = EL\_BUG\_PERCENT\_A;

g\_bug\_percent\_a[ tov\_OPotatoes ] = EL\_BUG\_PERCENT\_A;

g\_bug\_percent\_a[ tov\_FodderGrass ] = EL\_BUG\_PERCENT\_G\_A;

g\_bug\_percent\_a[ tov\_CloverGrassGrazed1 ] = EL\_BUG\_PERCENT\_G\_A;

g\_bug\_percent\_a[ tov\_CloverGrassGrazed2 ] = EL\_BUG\_PERCENT\_G\_A;

g\_bug\_percent\_a[ tov\_OCloverGrassGrazed1 ] = EL\_BUG\_PERCENT\_G\_A;

g\_bug\_percent\_a[ tov\_OCloverGrassGrazed2 ] = EL\_BUG\_PERCENT\_G\_A;

g\_bug\_percent\_a[ tov\_SpringBarley ] = EL\_BUG\_PERCENT\_SB\_A;

g\_bug\_percent\_a[ tov\_SpringBarleyPTreatment ] = EL\_BUG\_PERCENT\_SB\_A;

g\_bug\_percent\_a[ tov\_SpringBarleySKManagement ] = EL\_BUG\_PERCENT\_SB\_A;

g\_bug\_percent\_a[ tov\_WinterWheat ] = EL\_BUG\_PERCENT\_WW\_A;

g\_bug\_percent\_a[ tov\_WinterWheatShort ] = EL\_BUG\_PERCENT\_WW\_A;

g\_bug\_percent\_a[ tov\_SpringBarleySilage ] = EL\_BUG\_PERCENT\_SB\_A;

g\_bug\_percent\_a[ tov\_SpringBarleySeed ] = EL\_BUG\_PERCENT\_SB\_A;

g\_bug\_percent\_a[ tov\_OGrazingPigs ] = EL\_BUG\_PERCENT\_G\_A;

g\_bug\_percent\_a[ tov\_OCloverGrassSilage1 ] = EL\_BUG\_PERCENT\_G\_A;

g\_bug\_percent\_a[ tov\_SpringBarleyCloverGrass ] = EL\_BUG\_PERCENT\_SB\_A;

g\_bug\_percent\_a[ tov\_OSpringBarleyPigs ] = EL\_BUG\_PERCENT\_SB\_A;

g\_bug\_percent\_a[ tov\_OBarleyPeaCloverGrass ] = EL\_BUG\_PERCENT\_SB\_A;

g\_bug\_percent\_a[ tov\_OSpringBarley ] = EL\_BUG\_PERCENT\_SB\_A;

g\_bug\_percent\_a[ tov\_OSpringBarleyExt ] = EL\_BUG\_PERCENT\_SB\_A;

g\_bug\_percent\_a[ tov\_OSBarleySilage ] = EL\_BUG\_PERCENT\_SB\_A;

g\_bug\_percent\_a[ tov\_OWinterWheatUndersown ] = EL\_BUG\_PERCENT\_WW\_A;

g\_bug\_percent\_a[ tov\_OWinterWheatUndersownExt ] = EL\_BUG\_PERCENT\_WW\_A;

g\_bug\_percent\_a[ tov\_WinterRape ] = EL\_BUG\_PERCENT\_WR\_A;

g\_bug\_percent\_a[ tov\_SpringRape ] = EL\_BUG\_PERCENT\_WR\_A;

g\_bug\_percent\_a[ tov\_OWinterRape ] = EL\_BUG\_PERCENT\_WR\_A;

g\_bug\_percent\_a[ tov\_OWinterRye ] = EL\_BUG\_PERCENT\_WRy\_A;

g\_bug\_percent\_a[ tov\_OWinterBarley ] = EL\_BUG\_PERCENT\_A;

g\_bug\_percent\_a[ tov\_OWinterBarleyExt ] = EL\_BUG\_PERCENT\_A;

g\_bug\_percent\_a[ tov\_WinterBarley ] = EL\_BUG\_PERCENT\_A;

g\_bug\_percent\_a[ tov\_WinterRye ] = EL\_BUG\_PERCENT\_WRy\_A;

g\_bug\_percent\_a[ tov\_OFieldPeas ] = EL\_BUG\_PERCENT\_A;

g\_bug\_percent\_a[ tov\_OFieldPeasSilage ] = EL\_BUG\_PERCENT\_A;

g\_bug\_percent\_a[ tov\_OOats ] = EL\_BUG\_PERCENT\_A;

g\_bug\_percent\_a[ tov\_Oats ] = EL\_BUG\_PERCENT\_A;

g\_bug\_percent\_a[ tov\_FieldPeas ] = EL\_BUG\_PERCENT\_A;

g\_bug\_percent\_a[ tov\_SeedGrass1 ] = EL\_BUG\_PERCENT\_G\_A;

g\_bug\_percent\_a[ tov\_SeedGrass2 ] = EL\_BUG\_PERCENT\_G\_A;

g\_bug\_percent\_a[ tov\_OPermanentGrassGrazed ] = EL\_BUG\_PERCENT\_G\_A;

g\_bug\_percent\_a[ tov\_Setaside ] = EL\_BUG\_PERCENT\_Edges\_A;

g\_bug\_percent\_a[ tov\_OSetaside ] = EL\_BUG\_PERCENT\_Edges\_A;

g\_bug\_percent\_a[ tov\_PermanentSetaside ] = EL\_BUG\_PERCENT\_Edges\_A;

g\_bug\_percent\_a[ tov\_PermanentGrassLowYield ] = EL\_BUG\_PERCENT\_G\_A;

g\_bug\_percent\_a[ tov\_PermanentGrassGrazed ] = EL\_BUG\_PERCENT\_G\_A;

g\_bug\_percent\_a[ tov\_PermanentGrassTussocky ] = EL\_BUG\_PERCENT\_G\_A;

g\_bug\_percent\_a[ tov\_FodderBeet ] = EL\_BUG\_PERCENT\_A;

g\_bug\_percent\_a[ tov\_NaturalGrass ] = EL\_BUG\_PERCENT\_Edges\_A;

g\_bug\_percent\_a[ tov\_None ] = EL\_BUG\_PERCENT\_A;

g\_bug\_percent\_a[ tov\_NoGrowth ] = EL\_BUG\_PERCENT\_A;

g\_bug\_percent\_a[ tov\_OFirstYearDanger ] = EL\_BUG\_PERCENT\_A;

g\_bug\_percent\_a[ tov\_Triticale ] = EL\_BUG\_PERCENT\_A;

g\_bug\_percent\_a[ tov\_WWheatPControl ] = EL\_BUG\_PERCENT\_WW\_A;

g\_bug\_percent\_a[ tov\_WWheatPToxicControl ] = EL\_BUG\_PERCENT\_WW\_A;

g\_bug\_percent\_a[ tov\_WWheatPTreatment ] = EL\_BUG\_PERCENT\_WW\_A;

g\_bug\_percent\_a[ tov\_AgroChemIndustryCereal ] = EL\_BUG\_PERCENT\_WW\_A;

g\_bug\_percent\_a[ tov\_WinterWheatStrigling ] = EL\_BUG\_PERCENT\_WW\_A;

g\_bug\_percent\_a[ tov\_WinterWheatStriglingCulm ] = EL\_BUG\_PERCENT\_WW\_A;

g\_bug\_percent\_a[ tov\_WinterWheatStriglingSingle ] = EL\_BUG\_PERCENT\_WW\_A;

g\_bug\_percent\_a[ tov\_SpringBarleyCloverGrassStrigling ] = EL\_BUG\_PERCENT\_SB\_A;

g\_bug\_percent\_a[ tov\_SpringBarleyStrigling ] = EL\_BUG\_PERCENT\_SB\_A;

g\_bug\_percent\_a[ tov\_SpringBarleyStriglingSingle ] = EL\_BUG\_PERCENT\_SB\_A;

g\_bug\_percent\_a[ tov\_SpringBarleyStriglingCulm ] = EL\_BUG\_PERCENT\_SB\_A;

g\_bug\_percent\_a[ tov\_MaizeStrigling ] = EL\_BUG\_PERCENT\_A;

g\_bug\_percent\_a[ tov\_WinterRapeStrigling ] = EL\_BUG\_PERCENT\_WR\_A;

g\_bug\_percent\_a[ tov\_WinterRyeStrigling ] = EL\_BUG\_PERCENT\_WRy\_A;

g\_bug\_percent\_a[ tov\_WinterBarleyStrigling ] = EL\_BUG\_PERCENT\_A;

g\_bug\_percent\_a[ tov\_FieldPeasStrigling ] = EL\_BUG\_PERCENT\_A;

g\_bug\_percent\_a[ tov\_SpringBarleyPeaCloverGrassStrigling ] = EL\_BUG\_PERCENT\_SB\_A;

g\_bug\_percent\_a[ tov\_YoungForest ] = EL\_BUG\_PERCENT\_Edges\_A;

g\_bug\_percent\_b[ tov\_Carrots ] = EL\_BUG\_PERCENT\_B;

g\_bug\_percent\_b[ tov\_MaizeSilage ] = EL\_BUG\_PERCENT\_B;

g\_bug\_percent\_b[ tov\_OMaizeSilage ] = EL\_BUG\_PERCENT\_B;

g\_bug\_percent\_b[ tov\_Maize ] = EL\_BUG\_PERCENT\_B;

g\_bug\_percent\_b[ tov\_OCarrots ] = EL\_BUG\_PERCENT\_B;

g\_bug\_percent\_b[ tov\_Potatoes ] = EL\_BUG\_PERCENT\_B;

g\_bug\_percent\_b[ tov\_OPotatoes ] = EL\_BUG\_PERCENT\_B;

g\_bug\_percent\_b[ tov\_FodderGrass ] = EL\_BUG\_PERCENT\_G\_B;

g\_bug\_percent\_b[ tov\_CloverGrassGrazed1 ] = EL\_BUG\_PERCENT\_G\_B;

g\_bug\_percent\_b[ tov\_CloverGrassGrazed2 ] = EL\_BUG\_PERCENT\_G\_B;

g\_bug\_percent\_b[ tov\_OCloverGrassGrazed1 ] = EL\_BUG\_PERCENT\_G\_B;

g\_bug\_percent\_b[ tov\_OCloverGrassGrazed2 ] = EL\_BUG\_PERCENT\_G\_B;

g\_bug\_percent\_b[ tov\_SpringBarley ] = EL\_BUG\_PERCENT\_SB\_B;

g\_bug\_percent\_b[ tov\_SpringBarleyPTreatment ] = EL\_BUG\_PERCENT\_SB\_B;

g\_bug\_percent\_b[ tov\_SpringBarleySKManagement ] = EL\_BUG\_PERCENT\_SB\_B;

g\_bug\_percent\_b[ tov\_WinterWheat ] = EL\_BUG\_PERCENT\_WW\_B;

g\_bug\_percent\_b[ tov\_WinterWheatShort ] = EL\_BUG\_PERCENT\_WW\_B;

g\_bug\_percent\_b[ tov\_SpringBarleySilage ] = EL\_BUG\_PERCENT\_SB\_B;

g\_bug\_percent\_b[ tov\_SpringBarleySeed ] = EL\_BUG\_PERCENT\_SB\_B;

g\_bug\_percent\_b[ tov\_OGrazingPigs ] = EL\_BUG\_PERCENT\_G\_B;

g\_bug\_percent\_b[ tov\_OCloverGrassSilage1 ] = EL\_BUG\_PERCENT\_G\_B;

g\_bug\_percent\_b[ tov\_SpringBarleyCloverGrass ] = EL\_BUG\_PERCENT\_SB\_B;

g\_bug\_percent\_b[ tov\_OSpringBarleyPigs ] = EL\_BUG\_PERCENT\_SB\_B;

g\_bug\_percent\_b[ tov\_OBarleyPeaCloverGrass ] = EL\_BUG\_PERCENT\_SB\_B;

g\_bug\_percent\_b[ tov\_OSBarleySilage ] = EL\_BUG\_PERCENT\_SB\_B;

g\_bug\_percent\_b[ tov\_OSpringBarley ] = EL\_BUG\_PERCENT\_SB\_B;

g\_bug\_percent\_b[ tov\_OSpringBarleyExt ] = EL\_BUG\_PERCENT\_SB\_B;

g\_bug\_percent\_b[ tov\_OWinterWheatUndersown ] = EL\_BUG\_PERCENT\_WW\_B;

g\_bug\_percent\_b[ tov\_OWinterWheatUndersownExt ] = EL\_BUG\_PERCENT\_WW\_B;

g\_bug\_percent\_b[ tov\_WinterRape ] = EL\_BUG\_PERCENT\_WR\_B;

g\_bug\_percent\_b[ tov\_SpringRape ] = EL\_BUG\_PERCENT\_WR\_B;

g\_bug\_percent\_b[ tov\_OWinterRape ] = EL\_BUG\_PERCENT\_WR\_B;

g\_bug\_percent\_b[ tov\_OWinterRye ] = EL\_BUG\_PERCENT\_WRy\_B;

g\_bug\_percent\_b[ tov\_OWinterBarley ] = EL\_BUG\_PERCENT\_B;

g\_bug\_percent\_b[ tov\_OWinterBarleyExt ] = EL\_BUG\_PERCENT\_B;

g\_bug\_percent\_b[ tov\_WinterBarley ] = EL\_BUG\_PERCENT\_B;

g\_bug\_percent\_b[ tov\_WinterRye ] = EL\_BUG\_PERCENT\_WRy\_B;

g\_bug\_percent\_b[ tov\_OFieldPeas ] = EL\_BUG\_PERCENT\_B;

g\_bug\_percent\_b[ tov\_OFieldPeasSilage ] = EL\_BUG\_PERCENT\_B;

g\_bug\_percent\_b[ tov\_OOats ] = EL\_BUG\_PERCENT\_B;

g\_bug\_percent\_b[ tov\_Oats ] = EL\_BUG\_PERCENT\_B;

g\_bug\_percent\_b[ tov\_FieldPeas ] = EL\_BUG\_PERCENT\_B;

g\_bug\_percent\_b[ tov\_SeedGrass1 ] = EL\_BUG\_PERCENT\_G\_B;

g\_bug\_percent\_b[ tov\_SeedGrass2 ] = EL\_BUG\_PERCENT\_G\_B;

g\_bug\_percent\_b[ tov\_OPermanentGrassGrazed ] = EL\_BUG\_PERCENT\_G\_B;

g\_bug\_percent\_b[ tov\_Setaside ] = EL\_BUG\_PERCENT\_Edges\_B;

g\_bug\_percent\_b[ tov\_OSetaside ] = EL\_BUG\_PERCENT\_Edges\_B;

g\_bug\_percent\_b[ tov\_PermanentSetaside ] = EL\_BUG\_PERCENT\_Edges\_B;

g\_bug\_percent\_b[ tov\_PermanentGrassGrazed ] = EL\_BUG\_PERCENT\_G\_B;

g\_bug\_percent\_b[ tov\_PermanentGrassLowYield ] = EL\_BUG\_PERCENT\_G\_B;

g\_bug\_percent\_b[ tov\_PermanentGrassTussocky ] = EL\_BUG\_PERCENT\_G\_B;

g\_bug\_percent\_b[ tov\_FodderBeet ] = EL\_BUG\_PERCENT\_B;

g\_bug\_percent\_b[ tov\_NaturalGrass ] = EL\_BUG\_PERCENT\_Edges\_B;

g\_bug\_percent\_b[ tov\_None ] = EL\_BUG\_PERCENT\_B;

g\_bug\_percent\_b[ tov\_NoGrowth ] = EL\_BUG\_PERCENT\_B;

g\_bug\_percent\_b[ tov\_OFirstYearDanger ] = EL\_BUG\_PERCENT\_B;

g\_bug\_percent\_b[ tov\_Triticale ] = EL\_BUG\_PERCENT\_B;

g\_bug\_percent\_b[ tov\_WWheatPControl ] = EL\_BUG\_PERCENT\_WW\_B;

g\_bug\_percent\_b[ tov\_WWheatPToxicControl ] = EL\_BUG\_PERCENT\_WW\_B;

g\_bug\_percent\_b[ tov\_WWheatPTreatment ] = EL\_BUG\_PERCENT\_WW\_B;

g\_bug\_percent\_b[ tov\_AgroChemIndustryCereal ] = EL\_BUG\_PERCENT\_WW\_B;

g\_bug\_percent\_b[ tov\_WinterWheatStrigling ] = EL\_BUG\_PERCENT\_WW\_B;

g\_bug\_percent\_b[ tov\_WinterWheatStriglingCulm ] = EL\_BUG\_PERCENT\_WW\_B;

g\_bug\_percent\_b[ tov\_WinterWheatStriglingSingle ] = EL\_BUG\_PERCENT\_WW\_B;

g\_bug\_percent\_b[ tov\_SpringBarleyCloverGrassStrigling ] = EL\_BUG\_PERCENT\_SB\_B;

g\_bug\_percent\_b[ tov\_SpringBarleyStrigling ] = EL\_BUG\_PERCENT\_SB\_B;

g\_bug\_percent\_b[ tov\_SpringBarleyStriglingSingle ] = EL\_BUG\_PERCENT\_SB\_B;

g\_bug\_percent\_b[ tov\_SpringBarleyStriglingCulm ] = EL\_BUG\_PERCENT\_SB\_B;

g\_bug\_percent\_b[ tov\_MaizeStrigling ] = EL\_BUG\_PERCENT\_B;

g\_bug\_percent\_b[ tov\_WinterRapeStrigling ] = EL\_BUG\_PERCENT\_WR\_B;

g\_bug\_percent\_b[ tov\_WinterRyeStrigling ] = EL\_BUG\_PERCENT\_WRy\_B;

g\_bug\_percent\_b[ tov\_WinterBarleyStrigling ] = EL\_BUG\_PERCENT\_B;

g\_bug\_percent\_b[ tov\_FieldPeasStrigling ] = EL\_BUG\_PERCENT\_B;

g\_bug\_percent\_b[ tov\_SpringBarleyPeaCloverGrassStrigling ] = EL\_BUG\_PERCENT\_SB\_B;

g\_bug\_percent\_b[ tov\_YoungForest ] = EL\_BUG\_PERCENT\_Edges\_B;

g\_bug\_percent\_c[ tov\_Carrots ] = EL\_BUG\_PERCENT\_C;

g\_bug\_percent\_c[ tov\_MaizeSilage ] = EL\_BUG\_PERCENT\_C;

g\_bug\_percent\_c[ tov\_OMaizeSilage ] = EL\_BUG\_PERCENT\_C;

g\_bug\_percent\_c[ tov\_Maize ] = EL\_BUG\_PERCENT\_C;

g\_bug\_percent\_c[ tov\_OCarrots ] = EL\_BUG\_PERCENT\_C;

g\_bug\_percent\_c[ tov\_Potatoes ] = EL\_BUG\_PERCENT\_C;

g\_bug\_percent\_c[ tov\_OPotatoes ] = EL\_BUG\_PERCENT\_C;

g\_bug\_percent\_c[ tov\_FodderGrass ] = EL\_BUG\_PERCENT\_G\_C;

g\_bug\_percent\_c[ tov\_CloverGrassGrazed1 ] = EL\_BUG\_PERCENT\_G\_C;

g\_bug\_percent\_c[ tov\_CloverGrassGrazed2 ] = EL\_BUG\_PERCENT\_G\_C;

g\_bug\_percent\_c[ tov\_OCloverGrassGrazed1 ] = EL\_BUG\_PERCENT\_G\_C;

g\_bug\_percent\_c[ tov\_OCloverGrassGrazed2 ] = EL\_BUG\_PERCENT\_G\_C;

g\_bug\_percent\_c[ tov\_SpringBarley ] = EL\_BUG\_PERCENT\_SB\_C;

g\_bug\_percent\_c[ tov\_SpringBarleyPTreatment ] = EL\_BUG\_PERCENT\_SB\_C;

g\_bug\_percent\_c[ tov\_SpringBarleySKManagement ] = EL\_BUG\_PERCENT\_SB\_C;

g\_bug\_percent\_c[ tov\_WinterWheat ] = EL\_BUG\_PERCENT\_WW\_C;

g\_bug\_percent\_c[ tov\_WinterWheatShort ] = EL\_BUG\_PERCENT\_WW\_C;

g\_bug\_percent\_c[ tov\_SpringBarleySilage ] = EL\_BUG\_PERCENT\_SB\_C;

g\_bug\_percent\_c[ tov\_SpringBarleySeed ] = EL\_BUG\_PERCENT\_SB\_C;

g\_bug\_percent\_c[ tov\_OGrazingPigs ] = EL\_BUG\_PERCENT\_G\_C;

g\_bug\_percent\_c[ tov\_OCloverGrassSilage1 ] = EL\_BUG\_PERCENT\_G\_C;

g\_bug\_percent\_c[ tov\_SpringBarleyCloverGrass ] = EL\_BUG\_PERCENT\_SB\_C;

g\_bug\_percent\_c[ tov\_OSpringBarleyPigs ] = EL\_BUG\_PERCENT\_SB\_C;

g\_bug\_percent\_c[ tov\_OBarleyPeaCloverGrass ] = EL\_BUG\_PERCENT\_SB\_C;

g\_bug\_percent\_c[ tov\_OSBarleySilage ] = EL\_BUG\_PERCENT\_SB\_C;

g\_bug\_percent\_c[ tov\_OSpringBarley ] = EL\_BUG\_PERCENT\_SB\_C;

g\_bug\_percent\_c[ tov\_OSpringBarleyExt ] = EL\_BUG\_PERCENT\_SB\_C;

g\_bug\_percent\_c[ tov\_OWinterWheatUndersown ] = EL\_BUG\_PERCENT\_WW\_C;

g\_bug\_percent\_c[ tov\_OWinterWheatUndersownExt ] = EL\_BUG\_PERCENT\_WW\_C;

g\_bug\_percent\_c[ tov\_WinterRape ] = EL\_BUG\_PERCENT\_WR\_C;

g\_bug\_percent\_c[ tov\_SpringRape ] = EL\_BUG\_PERCENT\_WR\_C;

g\_bug\_percent\_c[ tov\_OWinterRape ] = EL\_BUG\_PERCENT\_WR\_C;

g\_bug\_percent\_c[ tov\_OWinterRye ] = EL\_BUG\_PERCENT\_WRy\_C;

g\_bug\_percent\_c[ tov\_OWinterBarley ] = EL\_BUG\_PERCENT\_C;

g\_bug\_percent\_c[ tov\_OWinterBarleyExt ] = EL\_BUG\_PERCENT\_C;

g\_bug\_percent\_c[ tov\_WinterBarley ] = EL\_BUG\_PERCENT\_C;

g\_bug\_percent\_c[ tov\_WinterRye ] = EL\_BUG\_PERCENT\_WRy\_C;

g\_bug\_percent\_c[ tov\_OFieldPeas ] = EL\_BUG\_PERCENT\_C;

g\_bug\_percent\_c[ tov\_OFieldPeasSilage ] = EL\_BUG\_PERCENT\_C;

g\_bug\_percent\_c[ tov\_OOats ] = EL\_BUG\_PERCENT\_C;

g\_bug\_percent\_c[ tov\_Oats ] = EL\_BUG\_PERCENT\_C;

g\_bug\_percent\_c[ tov\_FieldPeas ] = EL\_BUG\_PERCENT\_C;

g\_bug\_percent\_c[ tov\_SeedGrass1 ] = EL\_BUG\_PERCENT\_G\_C;

g\_bug\_percent\_c[ tov\_SeedGrass2 ] = EL\_BUG\_PERCENT\_G\_C;

g\_bug\_percent\_c[ tov\_OPermanentGrassGrazed ] = EL\_BUG\_PERCENT\_G\_C;

g\_bug\_percent\_c[ tov\_Setaside ] = EL\_BUG\_PERCENT\_Edges\_C;

g\_bug\_percent\_c[ tov\_OSetaside ] = EL\_BUG\_PERCENT\_Edges\_C;

g\_bug\_percent\_c[ tov\_PermanentSetaside ] = EL\_BUG\_PERCENT\_Edges\_C;

g\_bug\_percent\_c[ tov\_PermanentGrassGrazed ] = EL\_BUG\_PERCENT\_G\_C;

g\_bug\_percent\_c[ tov\_PermanentGrassLowYield ] = EL\_BUG\_PERCENT\_G\_C;

g\_bug\_percent\_c[ tov\_PermanentGrassTussocky ] = EL\_BUG\_PERCENT\_G\_C;

g\_bug\_percent\_c[ tov\_FodderBeet ] = EL\_BUG\_PERCENT\_C;

g\_bug\_percent\_c[ tov\_NaturalGrass ] = EL\_BUG\_PERCENT\_Edges\_C;

g\_bug\_percent\_c[ tov\_None ] = EL\_BUG\_PERCENT\_C;

g\_bug\_percent\_c[ tov\_NoGrowth ] = EL\_BUG\_PERCENT\_C;

g\_bug\_percent\_c[ tov\_OFirstYearDanger ] = EL\_BUG\_PERCENT\_C;

g\_bug\_percent\_c[ tov\_Triticale ] = EL\_BUG\_PERCENT\_C;

g\_bug\_percent\_c[ tov\_WWheatPControl ] = EL\_BUG\_PERCENT\_WW\_C;

g\_bug\_percent\_c[ tov\_WWheatPToxicControl ] = EL\_BUG\_PERCENT\_WW\_C;

g\_bug\_percent\_c[ tov\_WWheatPTreatment ] = EL\_BUG\_PERCENT\_WW\_C;

g\_bug\_percent\_c[ tov\_AgroChemIndustryCereal ] = EL\_BUG\_PERCENT\_WW\_C;

g\_bug\_percent\_c[ tov\_WinterWheatStrigling ] = EL\_BUG\_PERCENT\_WW\_C;

g\_bug\_percent\_c[ tov\_WinterWheatStriglingCulm ] = EL\_BUG\_PERCENT\_WW\_C;

g\_bug\_percent\_c[ tov\_WinterWheatStriglingSingle ] = EL\_BUG\_PERCENT\_WW\_C;

g\_bug\_percent\_c[ tov\_SpringBarleyCloverGrassStrigling ] = EL\_BUG\_PERCENT\_SB\_C;

g\_bug\_percent\_c[ tov\_SpringBarleyStrigling ] = EL\_BUG\_PERCENT\_SB\_C;

g\_bug\_percent\_c[ tov\_SpringBarleyStriglingSingle ] = EL\_BUG\_PERCENT\_SB\_C;

g\_bug\_percent\_c[ tov\_SpringBarleyStriglingCulm ] = EL\_BUG\_PERCENT\_SB\_C;

g\_bug\_percent\_c[ tov\_MaizeStrigling ] = EL\_BUG\_PERCENT\_C;

g\_bug\_percent\_c[ tov\_WinterRapeStrigling ] = EL\_BUG\_PERCENT\_WR\_C;

g\_bug\_percent\_c[ tov\_WinterRyeStrigling ] = EL\_BUG\_PERCENT\_WW\_C;

g\_bug\_percent\_c[ tov\_WinterBarleyStrigling ] = EL\_BUG\_PERCENT\_C;

g\_bug\_percent\_c[ tov\_FieldPeasStrigling ] = EL\_BUG\_PERCENT\_C;

g\_bug\_percent\_c[ tov\_SpringBarleyPeaCloverGrassStrigling ] = EL\_BUG\_PERCENT\_SB\_C;

g\_bug\_percent\_c[ tov\_YoungForest ] = EL\_BUG\_PERCENT\_Edges\_C;

g\_bug\_percent\_d[ tov\_Carrots ] = EL\_BUG\_PERCENT\_D;

g\_bug\_percent\_d[ tov\_Maize ] = EL\_BUG\_PERCENT\_D;

g\_bug\_percent\_d[ tov\_MaizeSilage ] = EL\_BUG\_PERCENT\_D;

g\_bug\_percent\_d[ tov\_OMaizeSilage ] = EL\_BUG\_PERCENT\_D;

g\_bug\_percent\_d[ tov\_OCarrots ] = EL\_BUG\_PERCENT\_D;

g\_bug\_percent\_d[ tov\_Potatoes ] = EL\_BUG\_PERCENT\_D;

g\_bug\_percent\_d[ tov\_OPotatoes ] = EL\_BUG\_PERCENT\_D;

g\_bug\_percent\_d[ tov\_FodderGrass ] = EL\_BUG\_PERCENT\_D;

g\_bug\_percent\_d[ tov\_CloverGrassGrazed1 ] = EL\_BUG\_PERCENT\_D;

g\_bug\_percent\_d[ tov\_CloverGrassGrazed2 ] = EL\_BUG\_PERCENT\_D;

g\_bug\_percent\_d[ tov\_OCloverGrassGrazed1 ] = EL\_BUG\_PERCENT\_D;

g\_bug\_percent\_d[ tov\_OCloverGrassGrazed2 ] = EL\_BUG\_PERCENT\_D;

g\_bug\_percent\_d[ tov\_SpringBarley ] = EL\_BUG\_PERCENT\_D;

g\_bug\_percent\_d[ tov\_SpringBarleyPTreatment ] = EL\_BUG\_PERCENT\_D;

g\_bug\_percent\_d[ tov\_SpringBarleySKManagement ] = EL\_BUG\_PERCENT\_D;

g\_bug\_percent\_d[ tov\_WinterWheat ] = EL\_BUG\_PERCENT\_D;

g\_bug\_percent\_d[ tov\_WinterWheatShort ] = EL\_BUG\_PERCENT\_D;

g\_bug\_percent\_d[ tov\_SpringBarleySilage ] = EL\_BUG\_PERCENT\_D;

g\_bug\_percent\_d[ tov\_SpringBarleySeed ] = EL\_BUG\_PERCENT\_D;

g\_bug\_percent\_d[ tov\_OGrazingPigs ] = EL\_BUG\_PERCENT\_D;

g\_bug\_percent\_d[ tov\_OCloverGrassSilage1 ] = EL\_BUG\_PERCENT\_D;

g\_bug\_percent\_d[ tov\_SpringBarleyCloverGrass ] = EL\_BUG\_PERCENT\_D;

g\_bug\_percent\_d[ tov\_OSpringBarleyPigs ] = EL\_BUG\_PERCENT\_D;

g\_bug\_percent\_d[ tov\_OBarleyPeaCloverGrass ] = EL\_BUG\_PERCENT\_D;

g\_bug\_percent\_d[ tov\_OSBarleySilage ] = EL\_BUG\_PERCENT\_D;

g\_bug\_percent\_d[ tov\_OSpringBarley ] = EL\_BUG\_PERCENT\_D;

g\_bug\_percent\_d[ tov\_OSpringBarleyExt ] = EL\_BUG\_PERCENT\_D;

g\_bug\_percent\_d[ tov\_OWinterWheatUndersown ] = EL\_BUG\_PERCENT\_D;

g\_bug\_percent\_d[ tov\_OWinterWheatUndersownExt ] = EL\_BUG\_PERCENT\_D;

g\_bug\_percent\_d[ tov\_WinterRape ] = EL\_BUG\_PERCENT\_D;

g\_bug\_percent\_d[ tov\_OWinterRape ] = EL\_BUG\_PERCENT\_D;

g\_bug\_percent\_d[ tov\_OWinterRye ] = EL\_BUG\_PERCENT\_D;

g\_bug\_percent\_d[ tov\_OWinterBarley ] = EL\_BUG\_PERCENT\_D;

g\_bug\_percent\_d[ tov\_OWinterBarleyExt ] = EL\_BUG\_PERCENT\_D;

g\_bug\_percent\_d[ tov\_WinterBarley ] = EL\_BUG\_PERCENT\_D;

g\_bug\_percent\_d[ tov\_WinterRye ] = EL\_BUG\_PERCENT\_D;

g\_bug\_percent\_d[ tov\_OFieldPeas ] = EL\_BUG\_PERCENT\_D;

g\_bug\_percent\_d[ tov\_OFieldPeasSilage ] = EL\_BUG\_PERCENT\_D;

g\_bug\_percent\_d[ tov\_OOats ] = EL\_BUG\_PERCENT\_D;

g\_bug\_percent\_d[ tov\_Oats ] = EL\_BUG\_PERCENT\_D;

g\_bug\_percent\_d[ tov\_FieldPeas ] = EL\_BUG\_PERCENT\_D;

g\_bug\_percent\_d[ tov\_SeedGrass1 ] = EL\_BUG\_PERCENT\_D;

g\_bug\_percent\_d[ tov\_SeedGrass2 ] = EL\_BUG\_PERCENT\_D;

g\_bug\_percent\_d[ tov\_OPermanentGrassGrazed ] = EL\_BUG\_PERCENT\_D;

g\_bug\_percent\_d[ tov\_Setaside ] = EL\_BUG\_PERCENT\_D;

g\_bug\_percent\_d[ tov\_OSetaside ] = EL\_BUG\_PERCENT\_D;

g\_bug\_percent\_d[ tov\_PermanentSetaside ] = EL\_BUG\_PERCENT\_D;

g\_bug\_percent\_d[ tov\_PermanentGrassLowYield ] = EL\_BUG\_PERCENT\_D;

g\_bug\_percent\_d[ tov\_PermanentGrassGrazed ] = EL\_BUG\_PERCENT\_D;

g\_bug\_percent\_d[ tov\_PermanentGrassTussocky ] = EL\_BUG\_PERCENT\_D;

g\_bug\_percent\_d[ tov\_FodderBeet ] = EL\_BUG\_PERCENT\_D;

g\_bug\_percent\_d[ tov\_NaturalGrass ] = EL\_BUG\_PERCENT\_D;

g\_bug\_percent\_d[ tov\_None ] = EL\_BUG\_PERCENT\_D;

g\_bug\_percent\_d[ tov\_NoGrowth ] = EL\_BUG\_PERCENT\_D;

g\_bug\_percent\_d[ tov\_OFirstYearDanger ] = EL\_BUG\_PERCENT\_D;

g\_bug\_percent\_d[ tov\_Triticale ] = EL\_BUG\_PERCENT\_D;

g\_bug\_percent\_d[ tov\_WWheatPControl ] = EL\_BUG\_PERCENT\_D;

g\_bug\_percent\_d[ tov\_WWheatPToxicControl ] = EL\_BUG\_PERCENT\_D;

g\_bug\_percent\_d[ tov\_WWheatPTreatment ] = EL\_BUG\_PERCENT\_D;

g\_bug\_percent\_d[ tov\_AgroChemIndustryCereal ] = EL\_BUG\_PERCENT\_D;

g\_bug\_percent\_d[ tov\_WinterWheatStrigling ] = EL\_BUG\_PERCENT\_D;

g\_bug\_percent\_d[ tov\_WinterWheatStriglingCulm ] = EL\_BUG\_PERCENT\_D;

g\_bug\_percent\_d[ tov\_WinterWheatStriglingSingle ] = EL\_BUG\_PERCENT\_D;

g\_bug\_percent\_d[ tov\_SpringBarleyCloverGrassStrigling ] = -EL\_BUG\_PERCENT\_D;

g\_bug\_percent\_d[ tov\_SpringBarleyStrigling ] = EL\_BUG\_PERCENT\_D;

g\_bug\_percent\_d[ tov\_SpringBarleyStriglingSingle ] = EL\_BUG\_PERCENT\_D;

g\_bug\_percent\_d[ tov\_SpringBarleyStriglingCulm ] = EL\_BUG\_PERCENT\_D;

g\_bug\_percent\_d[ tov\_MaizeStrigling ] = EL\_BUG\_PERCENT\_D;

g\_bug\_percent\_d[ tov\_WinterRapeStrigling ] = EL\_BUG\_PERCENT\_D;

g\_bug\_percent\_d[ tov\_WinterRyeStrigling ] = EL\_BUG\_PERCENT\_D;

g\_bug\_percent\_d[ tov\_WinterBarleyStrigling ] = EL\_BUG\_PERCENT\_D;

g\_bug\_percent\_d[ tov\_FieldPeasStrigling ] = EL\_BUG\_PERCENT\_D;

g\_bug\_percent\_d[ tov\_SpringBarleyPeaCloverGrassStrigling ] = EL\_BUG\_PERCENT\_D;

g\_bug\_percent\_d[ tov\_YoungForest ] = EL\_BUG\_PERCENT\_D;

}

|  |  |  |  |  |  |
| --- | --- | --- | --- | --- | --- |
| virtual VegElement::~VegElement | ( | void |  | ) |  |

inlinevirtual

{ ; };

---

## Member Function Documentation

|  |  |  |  |  |  |
| --- | --- | --- | --- | --- | --- |
| void VegElement::DoDevelopment | ( | void |  | ) |  |

virtual

Reimplemented from LE.

Reimplemented in OrchardGrass, OrchardBand, Orchard, ForestElement, UnsprayedFieldMargin, NaturalGrass, Scrub, GreenElement, RoadsideVerge, BeetleBank, HedgeBank, Hedges, and Field.

References cfg\_ele\_weedscaling, Calendar::Date(), ForceGrowthDevelopment(), g\_crops, g\_date, g\_landscape\_p, g\_weather, Weather::GetDDDegs(), CropData::GetHeightDiff(), CropData::GetLAgreenDiff(), CropData::GetLAtotalDiff(), m\_curve\_num, LE::m\_ddegs, m\_force\_growth, LE::m\_herbicidedelay, m\_LAgreen, m\_LAtotal, LE::m\_owner\_index, LE::m\_poly, m\_veg\_height, m\_veg\_phase, m\_weed\_biomass, m\_weed\_curve\_num, LE::m\_yddegs, RecalculateBugsNStuff(), Landscape::SupplyFarmIntensity(), and CfgFloat::value().

{

if ( m\_force\_growth ) {

ForceGrowthDevelopment();

RecalculateBugsNStuff();

return;

}

m\_yddegs = m\_ddegs;

m\_ddegs = m\_yddegs + g\_weather->GetDDDegs( g\_date->Date() );

double dLAG = g\_crops->GetLAgreenDiff( m\_ddegs, m\_yddegs, m\_curve\_num, m\_veg\_phase );

double dLAT = g\_crops->GetLAtotalDiff( m\_ddegs, m\_yddegs, m\_curve\_num, m\_veg\_phase );

double dHgt = g\_crops->GetHeightDiff( m\_ddegs, m\_yddegs, m\_curve\_num, m\_veg\_phase );

m\_LAgreen += dLAG;

if ( m\_LAgreen < 0.0 )

m\_LAgreen = 0.0;

m\_LAtotal += dLAT;

if ( m\_LAtotal < 0.0 )

m\_LAtotal = 0.0;

int fintensity = 0;

#ifdef \_\_EXTSHRINKSHEIGHT

if (this->m\_owner\_index != -1) { // This only works because only crops and similar structures have owners

fintensity = g\_landscape\_p->SupplyFarmIntensity(m\_poly);

if (fintensity==1) {

// 1 means extensive, so reduce vegetation height by 10%

dHgt \*=0.9;

}

}

#endif

m\_veg\_height += dHgt;

if ( m\_veg\_height < 0.0 ) m\_veg\_height = 0.0;

if ( m\_herbicidedelay == 0 ) {

double dWee = g\_crops->GetLAtotalDiff( m\_ddegs, m\_yddegs, m\_weed\_curve\_num, m\_veg\_phase );

m\_weed\_biomass += dWee \* cfg\_ele\_weedscaling.value()\* (1+fintensity);

}

if ( m\_weed\_biomass < 0.0 ) m\_weed\_biomass = 0.0;

RecalculateBugsNStuff();

}

|  |  |  |  |  |  |
| --- | --- | --- | --- | --- | --- |
| void VegElement::ForceGrowthDevelopment | ( | void |  | ) |  |

virtual

Reimplemented from LE.

References m\_force\_LAgreen, m\_force\_LAtotal, m\_force\_veg\_height, m\_LAgreen, m\_LAtotal, m\_veg\_height, and RecalculateBugsNStuff().

Referenced by DoDevelopment().

{

//if ( m\_herbicidedelay == 0 ) m\_weed\_biomass += m\_force\_Weed; // \*\*\*CJT\*\*\* 12th Sept 2008 - rather than force growth, weeds might be allowed to grow on their own

m\_LAgreen += m\_force\_LAgreen;

m\_LAtotal += m\_force\_LAtotal;

m\_veg\_height += m\_force\_veg\_height;

RecalculateBugsNStuff();

}

|  |  |  |  |  |  |
| --- | --- | --- | --- | --- | --- |
| void VegElement::ForceGrowthInitialize | ( | void |  | ) |  |

virtual

Reimplemented from LE.

References Calendar::DayInYear(), g\_crops, g\_date, CropData::GetStartValue(), janfirst, m\_curve\_num, m\_force\_growth, m\_force\_LAgreen, m\_force\_LAtotal, m\_force\_veg\_height, m\_force\_Weed, m\_LAgreen, m\_LAtotal, m\_veg\_height, m\_weed\_biomass, m\_weed\_curve\_num, marchfirst, RandomVegStartValues(), and CropData::StartValid().

Referenced by ForceGrowthSpringTest(), and ForceGrowthTest().

{

double LAgreen\_target;

double Weed\_target;

double LAtotal\_target;

double veg\_height\_target;

int next\_phase, daysleft;

// Figure out what our target phase is.

if ( g\_date->DayInYear() < g\_date->DayInYear( 3, 1 ) ) {

daysleft = g\_date->DayInYear( 1, 3 ) - g\_date->DayInYear();

next\_phase = marchfirst;

} else if ( g\_date->DayInYear() >= g\_date->DayInYear( 1, 11 ) ) {

daysleft = 365 - g\_date->DayInYear();

next\_phase = janfirst;

} else {

return;

}

if ( daysleft <= 0 )

// Uh! Oh! This really shouldn't happen.

return;

if ( !g\_crops->StartValid( m\_curve\_num, next\_phase ) ) {

// If no valid starting values for next phase, then

// preinitialize the random starting values! Ie. make the

// choice here and then do not choose another set come

// next phase transition, but use the values we already

// got at that point in time.

RandomVegStartValues( & LAtotal\_target, & LAgreen\_target, & veg\_height\_target, & Weed\_target );

} else {

Weed\_target = g\_crops->GetStartValue( m\_weed\_curve\_num, next\_phase, 0 );

LAgreen\_target = g\_crops->GetStartValue( m\_curve\_num, next\_phase, 0 );

LAtotal\_target = g\_crops->GetStartValue( m\_curve\_num, next\_phase, 1 );

veg\_height\_target = g\_crops->GetStartValue( m\_curve\_num, next\_phase, 2 );

}

m\_force\_growth = true;

m\_force\_Weed = ( Weed\_target - m\_weed\_biomass ) / ( double )daysleft;

m\_force\_LAgreen = ( LAgreen\_target - m\_LAgreen ) / ( double )daysleft;

m\_force\_LAtotal = ( LAtotal\_target - m\_LAtotal ) / ( double )daysleft;

m\_force\_veg\_height = ( veg\_height\_target - m\_veg\_height ) / ( double )daysleft;

}

|  |  |  |  |  |  |
| --- | --- | --- | --- | --- | --- |
| void VegElement::ForceGrowthSpringTest | ( | void |  | ) |  |

protected

References ForceGrowthInitialize(), g\_crops, CropData::GetHeightDiff(), CropData::GetLAgreenDiff(), CropData::GetLAtotalDiff(), janfirst, m\_curve\_num, m\_vege\_type, tov\_NaturalGrass, and tov\_PermanentSetaside.

Referenced by SetGrowthPhase().

{

// Unless we are on the list of special growth curves where we have worked

// out vegetation declines, then

// check if there are any positive growth differentials in the curve

// for the first two months of the year. Do nothing if there is.

switch (m\_vege\_type) {

case tov\_NaturalGrass:

case tov\_PermanentSetaside:

return;

default:

if ( g\_crops->GetLAgreenDiff( 90000.0, 0.0, m\_curve\_num, janfirst ) > 0.001

|| g\_crops->GetLAtotalDiff( 90000.0, 0.0, m\_curve\_num, janfirst ) > 0.001

|| g\_crops->GetHeightDiff( 90000.0, 0.0, m\_curve\_num, janfirst ) > 0.001 ) {

return;

}

}

// No growth, force it.

ForceGrowthInitialize();

}

|  |  |  |  |  |  |
| --- | --- | --- | --- | --- | --- |
| void VegElement::ForceGrowthTest | ( | void |  | ) |  |

virtual

Reimplemented from LE.

References Calendar::DayInYear(), ForceGrowthInitialize(), g\_date, and m\_force\_growth.

Referenced by ReduceVeg(), ReduceVeg\_Extended(), and ZeroVeg().

{

// Called whenever the farmer does something 'destructive' to a

// field, that reduced the vegetaion.

if ( g\_date->DayInYear() >= g\_date->DayInYear( 1, 11 )

|| ( g\_date->DayInYear() < g\_date->DayInYear( 1, 3 ) && m\_force\_growth ) ) {

ForceGrowthInitialize();

}

}

|  |  |  |  |  |  |
| --- | --- | --- | --- | --- | --- |
| virtual double VegElement::GetDeadBiomass | ( | void |  | ) |  |

inlinevirtual

Reimplemented from LE.

References m\_dead\_biomass.

{ return m\_dead\_biomass; }

|  |  |  |  |  |  |
| --- | --- | --- | --- | --- | --- |
| virtual double VegElement::GetDigestability | ( | void |  | ) |  |

inlinevirtual

Reimplemented from LE.

References m\_digestability.

{ return m\_digestability; }

|  |  |  |  |  |  |
| --- | --- | --- | --- | --- | --- |
| virtual double VegElement::GetGreenBiomass | ( | void |  | ) |  |

inlinevirtual

Reimplemented from LE.

References m\_green\_biomass.

{ return m\_green\_biomass; }

|  |  |  |  |  |  |
| --- | --- | --- | --- | --- | --- |
| double VegElement::GetInsectPop | ( | void |  | ) |  |

inlinevirtual

Reimplemented from LE.

References m\_insect\_pop.

{

return m\_insect\_pop;

}

|  |  |  |  |  |  |
| --- | --- | --- | --- | --- | --- |
| virtual double VegElement::GetLAGreen | ( | void |  | ) |  |

inlinevirtual

Reimplemented from LE.

References m\_LAgreen.

{ return m\_LAgreen; }

|  |  |  |  |  |  |
| --- | --- | --- | --- | --- | --- |
| virtual double VegElement::GetLATotal | ( | void |  | ) |  |

inlinevirtual

Reimplemented from LE.

References m\_LAtotal.

{ return m\_LAtotal; }

|  |  |  |  |  |  |
| --- | --- | --- | --- | --- | --- |
| virtual bool VegElement::GetSkScrapes | ( | void |  | ) |  |

inlinevirtual

Reimplemented from LE.

References LE::m\_skylarkscrapes.

{ return m\_skylarkscrapes; }

|  |  |  |  |  |  |
| --- | --- | --- | --- | --- | --- |
| virtual double VegElement::GetVegBiomass | ( | void |  | ) |  |

inlinevirtual

Reimplemented from LE.

References m\_veg\_biomass.

{ return m\_veg\_biomass; }

|  |  |  |  |  |  |
| --- | --- | --- | --- | --- | --- |
| virtual double VegElement::GetVegCover | ( | void |  | ) |  |

inlinevirtual

Reimplemented from LE.

References m\_veg\_cover.

{ return m\_veg\_cover; }

|  |  |  |  |  |  |
| --- | --- | --- | --- | --- | --- |
| virtual int VegElement::GetVegDensity | ( | void |  | ) |  |

inlinevirtual

Reimplemented from LE.

References m\_veg\_density.

{ return m\_veg\_density; }

|  |  |  |  |  |  |
| --- | --- | --- | --- | --- | --- |
| virtual double VegElement::GetVegHeight | ( | void |  | ) |  |

inlinevirtual

Reimplemented from LE.

References m\_veg\_height.

{ return m\_veg\_height; }

|  |  |  |  |  |  |
| --- | --- | --- | --- | --- | --- |
| virtual bool VegElement::GetVegPatchy | ( | void |  | ) |  |

inlinevirtual

Reimplemented from LE.

References m\_veg\_patchy.

{ return m\_veg\_patchy; }

|  |  |  |  |  |  |
| --- | --- | --- | --- | --- | --- |
| virtual TTypesOfVegetation VegElement::GetVegType | ( | void |  | ) |  |

inlinevirtual

Reimplemented from LE.

References m\_vege\_type.

{ return m\_vege\_type; }

|  |  |  |  |  |  |
| --- | --- | --- | --- | --- | --- |
| virtual double VegElement::GetWeedBiomass | ( | void |  | ) |  |

inlinevirtual

Reimplemented from LE.

References m\_weed\_biomass.

{ return m\_weed\_biomass; }

|  |  |  |  |  |  |
| --- | --- | --- | --- | --- | --- |
| virtual void VegElement::Insecticide | ( | double | *a\_fraction* | ) |  |

inlinevirtual

Reimplemented from LE.

References LE::m\_days\_since\_insecticide\_spray, and m\_insect\_pop.

{

m\_insect\_pop \*= a\_fraction;

m\_days\_since\_insecticide\_spray=28;

}

|  |  |  |  |  |  |
| --- | --- | --- | --- | --- | --- |
| void VegElement::InsectMortality | ( | double | *a\_fraction* | ) |  |

virtual

Reimplemented from LE.

References m\_insect\_pop.

{

m\_insect\_pop \*= a\_fraction;

}

|  |  |  |  |
| --- | --- | --- | --- |
| void VegElement::RandomVegStartValues | ( | double \* | *a\_LAtotal*, |
|  |  | double \* | *a\_LAgreen*, |
|  |  | double \* | *a\_veg\_height*, |
|  |  | double \* | *a\_weed\_biomass* |
|  | ) |  |  |

protected

References EL\_VEG\_HEIGHTSCALE, EL\_VEG\_START\_LAIT, and random().

Referenced by ForceGrowthInitialize(), and SetGrowthPhase().

{

\* a\_LAtotal = EL\_VEG\_START\_LAIT \* ( ( ( ( double )( random( 21 ) - 10 ) ) / 100.0 ) + 1.0 ); // +/- 10%

\* a\_LAgreen = \* a\_LAtotal / 4.0;

\* a\_veg\_height = \* a\_LAgreen \* EL\_VEG\_HEIGHTSCALE;

\* a\_weed\_biomass = \* a\_LAgreen \* 0.1; // 10% weeds by biomass

}

|  |  |  |  |  |  |
| --- | --- | --- | --- | --- | --- |
| void VegElement::ReadBugPercentageFile | ( | void |  | ) |  |

protected

References FloatToDouble(), g\_bug\_percent\_a, g\_bug\_percent\_b, g\_bug\_percent\_c, g\_bug\_percent\_d, g\_msg, g\_weed\_percent, l\_el\_bug\_percentage\_file(), m\_ifile, tov\_Undefined, CfgStr::value(), MapErrorMsg::Warn(), and WARN\_FILE.

{

m\_ifile=fopen(l\_el\_bug\_percentage\_file.value(), "r" );

if ( !m\_ifile ) {

g\_msg->Warn( WARN\_FILE, "CropData::ReadBugPercentageFile(): Unable to open file", l\_el\_bug\_percentage\_file.value() );

exit( 1 );

}

for ( int i = 0; i < tov\_Undefined; i++ ) {

int vegnum;

// \*\*cjt\*\* modified 31/01/2004

float weedpercent, bugpercent\_a, bugpercent\_b, bugpercent\_c, bugpercent\_d;

if ( 2 != fscanf( m\_ifile, "%d %f %f %f %f %f", & vegnum, & weedpercent, & bugpercent\_a, & bugpercent\_b,

& bugpercent\_c, & bugpercent\_d ) ) {

char vegnums[ 20 ];

sprintf( vegnums, "%d", tov\_Undefined );

g\_msg->Warn( WARN\_FILE,

"VegElement::ReadBugPercentageFile(): Unable to read"

" sufficient number of int/double pairs from bug percentage file."" Lines expected:", vegnums );

exit( 1 );

}

FloatToDouble( g\_weed\_percent[ vegnum ], weedpercent);

FloatToDouble( g\_bug\_percent\_a[ vegnum ] , bugpercent\_a);

FloatToDouble( g\_bug\_percent\_b[ vegnum ] , bugpercent\_b);

FloatToDouble( g\_bug\_percent\_c[ vegnum ] , bugpercent\_c);

FloatToDouble( g\_bug\_percent\_d[ vegnum ] , bugpercent\_d);

}

fclose( m\_ifile );

}

|  |  |  |  |  |  |
| --- | --- | --- | --- | --- | --- |
| void VegElement::RecalculateBugsNStuff | ( | void |  | ) |  |

protectedvirtual

Reimplemented from LE.

References c\_SolarConversion, g\_biomass\_scale, g\_bug\_percent\_a, g\_bug\_percent\_b, g\_bug\_percent\_c, g\_landscape\_p, LE::m\_days\_since\_insecticide\_spray, m\_dead\_biomass, m\_digestability, m\_green\_biomass, m\_insect\_pop, m\_LAgreen, m\_LAtotal, m\_newgrowthsum, m\_newoldgrowthindex, m\_oldLAtotal, m\_oldnewgrowth, LE::m\_owner\_index, LE::m\_poly, m\_veg\_biomass, m\_veg\_cover, m\_veg\_density, m\_veg\_height, m\_vege\_type, m\_weed\_biomass, Landscape::SupplyFarmIntensity(), Landscape::SupplyGlobalRadiation(), Landscape::SupplyTemp(), tov\_Maize, tov\_MaizeSilage, tov\_MaizeStrigling, tov\_NoGrowth, tov\_None, tov\_OFirstYearDanger, tov\_OMaizeSilage, tov\_OPotatoes, tov\_Potatoes, and tov\_PotatoesIndustry.

Referenced by RoadsideVerge::Cutting(), DoDevelopment(), ForceGrowthDevelopment(), ReduceVeg(), ReduceVeg\_Extended(), SetVegHeight(), and ZeroVeg().

{

double newgrowth=0;

m\_veg\_cover = 1.0 - ( exp( m\_LAtotal \* -0.6 ) ); // Beer's Law to give cover

double usefull\_veg\_cover = 1.0 - ( exp( m\_LAgreen \* -0.4 ) ); // This is used to calc growth rate

// Need gloabal radiation today

double glrad = g\_landscape\_p->SupplyGlobalRadiation();

// This is different for maize (a C4 plant)

int ptype;

if (( m\_vege\_type == tov\_Maize ) || ( m\_vege\_type == tov\_OMaizeSilage ) || (m\_vege\_type == tov\_MaizeSilage) || (m\_vege\_type == tov\_MaizeStrigling)) ptype = 1; else ptype = 0;

int index = ( int )floor( 0.5 + g\_landscape\_p->SupplyTemp() ) + 30; // There are 30 negative temps

double radconv = c\_SolarConversion[ ptype ] [ index ];

if ( m\_LAtotal >= m\_oldLAtotal ) {

// we are in positive growth so grow depending on our equation

newgrowth = usefull\_veg\_cover \* glrad \* radconv \* g\_biomass\_scale[ m\_vege\_type ];

if (this->m\_owner\_index != -1) { // This only works because only crops and similar structures have owners

int fintensity = g\_landscape\_p->SupplyFarmIntensity(m\_poly);

if (fintensity==1) {

// 1 means extensive, so reduce vegetation biomass by 20%

// NB this cannot be used with extensive crop types otherwise you get an additional 20% reduction

// This way of doing things provides a quick and dirty general effect.

m\_veg\_biomass += newgrowth \* 0.8;

} else m\_veg\_biomass += newgrowth;

} else m\_veg\_biomass += newgrowth;

} else {

// Negative growth - so shrink proportional to the loss in LAI Total

if ( m\_oldLAtotal > 0 ) {

m\_veg\_biomass \*= ( m\_LAtotal / m\_oldLAtotal );

}

}

// Remaining thing to do for the hare (& others?) is to calculate mean vegetation digestability

// This is a 16-day running average of the amount of new growth per day divided by veg biomass

// With a minimum value of 0.5

++m\_newoldgrowthindex &= 31;

if (m\_veg\_biomass>0 ) {

switch (m\_vege\_type) {

case tov\_NoGrowth:

case tov\_None:

case tov\_OFirstYearDanger:

m\_digestability=0.0;

break;

case tov\_OPotatoes:

case tov\_Maize:

case tov\_MaizeSilage:

case tov\_OMaizeSilage:

case tov\_MaizeStrigling:

case tov\_Potatoes:

case tov\_PotatoesIndustry:

m\_digestability=0.5;

break;

default:

//m\_oldnewgrowth[m\_newoldgrowthindex]=(newgrowth/m\_veg\_biomass);

m\_oldnewgrowth[m\_newoldgrowthindex]=(newgrowth);

m\_newgrowthsum=0.0;

for (int i=0; i<32; i++) {

m\_newgrowthsum+=m\_oldnewgrowth[i];

}

m\_digestability = m\_newgrowthsum/m\_veg\_biomass;

m\_digestability+=0.5;

if (m\_digestability>0.8) m\_digestability=0.8;

}

}

else {

m\_oldnewgrowth[m\_newoldgrowthindex] = 0.0;

m\_newgrowthsum=0.0;

for (int i=0; i<32; i++) {

m\_newgrowthsum+=m\_oldnewgrowth[i];

}

m\_digestability = 0;

}

// NB The m\_weed\_biomass is calculated directly from the curve in Curves.pre

// rather than going through the rigmorole of converting leaf-area index

// The insect calculation part

// Bugmass = a + b(biomass) + c(height)

double temp\_bugmass = //g\_bug\_percent\_d[ m\_vege\_type ] // This was used as a scaler - now not used

g\_bug\_percent\_a[ m\_vege\_type ] + ( ( m\_veg\_biomass + m\_weed\_biomass ) \* g\_bug\_percent\_b[ m\_vege\_type ] )

+ ( m\_veg\_height \* g\_bug\_percent\_c[ m\_vege\_type ] );

// Set a minimum value (regressions will otherwise sometimes give a -ve value

if ( temp\_bugmass < 0.05 ) temp\_bugmass = 0.05;

// Now need to check for deviations caused by management

// First spot the deviation - this is easy because the only deviation that does

// not affect the vegetation too is insecticide spraying

if ( m\_days\_since\_insecticide\_spray > 0 ) {

// Need to change insects still, so grow towards the target, but only when 21 days from zero effect

if (m\_days\_since\_insecticide\_spray<21) m\_insect\_pop += (temp\_bugmass-m\_insect\_pop)/m\_days\_since\_insecticide\_spray;

m\_days\_since\_insecticide\_spray--;

}

else {

m\_insect\_pop = temp\_bugmass;

}

m\_veg\_density = (int) (floor( 0.5 + ( m\_veg\_biomass / ( 1 + m\_veg\_height ) ) ));

if ( m\_veg\_density > 100 ) m\_veg\_density = 100; // to stop array bounds problems

// EcoSol Added 6/10/2007

// So we can ask about the green vs dead biomass

m\_green\_biomass = m\_veg\_biomass \* (m\_LAgreen/(m\_LAtotal));

m\_dead\_biomass = m\_veg\_biomass-m\_green\_biomass;

// End EcoSol code

}

|  |  |  |  |  |  |
| --- | --- | --- | --- | --- | --- |
| void VegElement::ReduceVeg | ( | double | *a\_reduc* | ) |  |

virtual

Reimplemented from LE.

References ForceGrowthTest(), m\_LAgreen, m\_LAtotal, m\_veg\_biomass, m\_veg\_height, m\_weed\_biomass, and RecalculateBugsNStuff().

Referenced by OrchardBand::DoDevelopment().

{

m\_LAgreen \*= a\_reduc;

m\_LAtotal \*= a\_reduc;

m\_veg\_height \*= a\_reduc;

m\_veg\_biomass \*= a\_reduc;

m\_weed\_biomass \*= a\_reduc;

ForceGrowthTest();

RecalculateBugsNStuff();

}

|  |  |  |  |  |  |
| --- | --- | --- | --- | --- | --- |
| void VegElement::ReduceVeg\_Extended | ( | double | *a\_reduc* | ) |  |

virtual

Reimplemented from LE.

References Calendar::DayInYear(), EL\_GROWTH\_DATE\_MAGIC, EL\_GROWTH\_DAYDEG\_MAGIC, EL\_GROWTH\_PHASE\_SHIFT\_LEVEL, ForceGrowthTest(), g\_date, harvest1, LE::m\_ddegs, m\_forced\_phase\_shift, m\_LAgreen, m\_LAtotal, m\_veg\_biomass, m\_veg\_height, m\_weed\_biomass, LE::m\_yddegs, RecalculateBugsNStuff(), and SetGrowthPhase().

{

m\_LAgreen \*= a\_reduc;

m\_LAtotal \*= a\_reduc;

m\_veg\_height \*= a\_reduc;

m\_veg\_biomass \*= a\_reduc;

m\_weed\_biomass \*= a\_reduc;

if ( a\_reduc < EL\_GROWTH\_PHASE\_SHIFT\_LEVEL ) {

m\_yddegs = 0.0;

m\_ddegs = EL\_GROWTH\_DAYDEG\_MAGIC;

}

if ( g\_date->DayInYear() >= EL\_GROWTH\_DATE\_MAGIC && a\_reduc < EL\_GROWTH\_PHASE\_SHIFT\_LEVEL && !m\_forced\_phase\_shift ) {

SetGrowthPhase( harvest1 );

m\_forced\_phase\_shift = true;

}

ForceGrowthTest();

RecalculateBugsNStuff();

}

|  |  |  |  |  |  |
| --- | --- | --- | --- | --- | --- |
| virtual void VegElement::ReduceWeedBiomass | ( | double | *a\_fraction* | ) |  |

inlinevirtual

Reimplemented from LE.

References m\_weed\_biomass.

{

m\_weed\_biomass \*= a\_fraction;

}

|  |  |  |  |
| --- | --- | --- | --- |
| void VegElement::SetCropData | ( | double | *a\_veg\_height*, |
|  |  | double | *a\_LAtotal*, |
|  |  | double | *a\_LAgreen*, |
|  |  | TTypesOfVegetation | *a\_veg*, |
|  |  | double | *a\_cover*, |
|  |  | bool | *a\_grazed* |
|  | ) |  |  |

virtual

Reimplemented from LE.

References LE::m\_cattle\_grazing, m\_LAgreen, m\_LAtotal, m\_veg\_cover, m\_veg\_height, and m\_vege\_type.

{

m\_veg\_height = a\_veg\_height;

m\_LAtotal = a\_LAtotal;

m\_LAgreen = a\_LAgreen;

m\_vege\_type = a\_veg;

m\_veg\_cover = a\_cover;

m\_cattle\_grazing = a\_grazed;

}

|  |  |  |  |
| --- | --- | --- | --- |
| void VegElement::SetCropDataAll | ( | double | *a\_veg\_height*, |
|  |  | double | *a\_biomass*, |
|  |  | double | *a\_LAtotal*, |
|  |  | double | *a\_LAgreen*, |
|  |  | TTypesOfVegetation | *a\_veg*, |
|  |  | double | *a\_wb*, |
|  |  | double | *a\_cover*, |
|  |  | bool | *a\_grazed*, |
|  |  | double | *a\_ins*, |
|  |  | bool | *a\_patchy*, |
|  |  | double | *a\_dens* |
|  | ) |  |  |

virtual

Reimplemented from LE.

References LE::m\_cattle\_grazing, m\_insect\_pop, m\_LAgreen, m\_LAtotal, m\_veg\_biomass, m\_veg\_cover, m\_veg\_density, m\_veg\_height, m\_veg\_patchy, m\_vege\_type, and m\_weed\_biomass.

{

m\_veg\_height = a\_veg\_height;

m\_veg\_biomass = a\_biomass;

m\_LAtotal = a\_LAtotal;

m\_LAgreen = a\_LAgreen;

m\_vege\_type = a\_veg;

m\_weed\_biomass = a\_wb;

m\_veg\_cover = a\_cover;

m\_cattle\_grazing = a\_grazed;

m\_insect\_pop = a\_ins;

m\_veg\_density = (int) a\_dens;

m\_veg\_patchy = a\_patchy;

}

|  |  |  |  |  |  |
| --- | --- | --- | --- | --- | --- |
| void VegElement::SetGrowthPhase | ( | int | *a\_phase* | ) |  |

virtual

Reimplemented from LE.

References Calendar::Date(), ForceGrowthSpringTest(), g\_crops, g\_date, g\_weather, Weather::GetDDDegs(), CropData::GetStartValue(), janfirst, m\_curve\_num, LE::m\_ddegs, m\_force\_growth, m\_forced\_phase\_shift, m\_LAgreen, m\_LAtotal, m\_veg\_height, m\_veg\_phase, m\_vege\_type, m\_weed\_biomass, LE::m\_yddegs, RandomVegStartValues(), CropData::StartValid(), and tov\_PermanentSetaside.

Referenced by Orchard::Cutting(), OrchardGrass::Cutting(), ReduceVeg\_Extended(), and VegElement().

{

if ( m\_vege\_type == tov\_PermanentSetaside && a\_phase != janfirst )

return;

if ( a\_phase == janfirst )

m\_forced\_phase\_shift = false;

if ( g\_crops->StartValid( m\_curve\_num, a\_phase ) ) {

m\_LAgreen = g\_crops->GetStartValue( m\_curve\_num, a\_phase, 0 );

m\_LAtotal = g\_crops->GetStartValue( m\_curve\_num, a\_phase, 1 );

m\_veg\_height = g\_crops->GetStartValue( m\_curve\_num, a\_phase, 2 );

} else if ( !m\_force\_growth ) {

// If we are in forced growth mode (which is very likely),

// then do not choose a new set of starting values, as we have

// already calculated our way to a reasonable set of values.

RandomVegStartValues( & m\_LAtotal, & m\_LAgreen, & m\_veg\_height, & m\_weed\_biomass );

}

m\_veg\_phase = a\_phase;

m\_yddegs = 0.0;

m\_ddegs = g\_weather->GetDDDegs( g\_date->Date() );

m\_force\_growth = false;

if ( m\_veg\_phase == janfirst ) {

// For some growth curves there is no growth in the first

// two months of the year. This will more likely than

// not cause a discontinuous jump in the growth curves

// come March first. ForceGrowthSpringTest() tries

// to avoid that by checking for positive growth values

// for the January growth phase. If none are found, then

// it initializes a forced growth/transition to the March

// 1st starting values.

ForceGrowthSpringTest();

}

}

|  |  |  |  |  |  |
| --- | --- | --- | --- | --- | --- |
| virtual void VegElement::SetInsectPop | ( | double | *insects* | ) |  |

inlinevirtual

Reimplemented from LE.

References m\_insect\_pop.

{ m\_insect\_pop=insects; }

|  |  |  |  |
| --- | --- | --- | --- |
| virtual void VegElement::SetVegHeight | ( | double | *a\_veg\_height*, |
|  |  | double | *a\_LAtotal*, |
|  |  | double | *a\_LAgreen*, |
|  |  | double | *a\_WeedBiomass* |
|  | ) |  |  |

inlinevirtual

Reimplemented from LE.

References m\_LAgreen, m\_LAtotal, m\_veg\_height, m\_weed\_biomass, and RecalculateBugsNStuff().

{

m\_veg\_height = a\_veg\_height;

m\_LAtotal = a\_LAtotal;

m\_LAgreen = a\_LAgreen;

m\_weed\_biomass = a\_WeedBiomass;

RecalculateBugsNStuff();

}

|  |  |  |  |  |  |
| --- | --- | --- | --- | --- | --- |
| virtual void VegElement::SetVegPatchy | ( | bool | *p* | ) |  |

inlinevirtual

Reimplemented from LE.

References m\_veg\_patchy.

Referenced by VegElement().

{ m\_veg\_patchy=p; }

|  |  |  |  |
| --- | --- | --- | --- |
| virtual void VegElement::SetVegType | ( | TTypesOfVegetation | *a\_vege\_type*, |
|  |  | TTypesOfVegetation | *a\_weed\_type* |
|  | ) |  |  |

inlinevirtual

Reimplemented from LE.

References g\_crops, m\_curve\_num, m\_vege\_type, m\_weed\_curve\_num, tov\_Undefined, and CropData::VegTypeToCurveNum().

{

m\_vege\_type = a\_vege\_type;

m\_curve\_num = g\_crops->VegTypeToCurveNum( a\_vege\_type );

// -1 is used as a signal not to change the weed type

// this is because it may be specific to that field

if (a\_weed\_type!=tov\_Undefined) m\_weed\_curve\_num = a\_weed\_type;

}

|  |  |  |  |  |
| --- | --- | --- | --- | --- |
| virtual void VegElement::StoreLAItotal | ( |  | ) |  |

inlinevirtual

Reimplemented from LE.

References m\_LAtotal, and m\_oldLAtotal.

{ m\_oldLAtotal = m\_LAtotal; }

|  |  |  |  |  |  |
| --- | --- | --- | --- | --- | --- |
| virtual void VegElement::ToggleCattleGrazing | ( | void |  | ) |  |

inlinevirtual

Reimplemented from LE.

References LE::m\_cattle\_grazing.

{ m\_cattle\_grazing = !m\_cattle\_grazing; }

|  |  |  |  |  |  |
| --- | --- | --- | --- | --- | --- |
| virtual void VegElement::TogglePigGrazing | ( | void |  | ) |  |

inlinevirtual

Reimplemented from LE.

References LE::m\_pig\_grazing.

{ m\_pig\_grazing = !m\_pig\_grazing; }

|  |  |  |  |  |  |
| --- | --- | --- | --- | --- | --- |
| void VegElement::ZeroVeg | ( | void |  | ) |  |

virtual

Reimplemented from LE.

References ForceGrowthTest(), m\_LAgreen, m\_LAtotal, m\_veg\_biomass, m\_veg\_cover, m\_veg\_height, m\_weed\_biomass, and RecalculateBugsNStuff().

{

m\_LAgreen = 0.0;

m\_LAtotal = 0.0;

m\_veg\_height = 0.0;

m\_veg\_cover = 0.0;

m\_veg\_biomass = 0.0;

m\_weed\_biomass = 0.0;

ForceGrowthTest();

RecalculateBugsNStuff();

}

---

## Member Data Documentation

int VegElement::m\_curve\_num

protected

Referenced by BeetleBank::BeetleBank(), DoDevelopment(), FieldBoundary::FieldBoundary(), ForceGrowthInitialize(), ForceGrowthSpringTest(), Heath::Heath(), HedgeBank::HedgeBank(), Hedges::Hedges(), Marsh::Marsh(), NaturalGrass::NaturalGrass(), Orchard::Orchard(), OrchardBand::OrchardBand(), OrchardGrass::OrchardGrass(), PermanentSetaside::PermanentSetaside(), SetGrowthPhase(), SetVegType(), UnsprayedFieldMargin::UnsprayedFieldMargin(), VegElement(), and YoungForest::YoungForest().

double VegElement::m\_dead\_biomass

protected

Referenced by GetDeadBiomass(), and RecalculateBugsNStuff().

double VegElement::m\_digestability

protected

Referenced by NaturalGrass::DoDevelopment(), GetDigestability(), PermanentSetaside::PermanentSetaside(), PermPastureTussocky::PermPastureTussocky(), RecalculateBugsNStuff(), and VegElement().

bool VegElement::m\_force\_growth

protected

Referenced by DoDevelopment(), ForceGrowthInitialize(), ForceGrowthTest(), SetGrowthPhase(), and VegElement().

double VegElement::m\_force\_LAgreen

protected

Referenced by ForceGrowthDevelopment(), and ForceGrowthInitialize().

double VegElement::m\_force\_LAtotal

protected

Referenced by ForceGrowthDevelopment(), and ForceGrowthInitialize().

double VegElement::m\_force\_veg\_height

protected

Referenced by ForceGrowthDevelopment(), and ForceGrowthInitialize().

double VegElement::m\_force\_Weed

protected

Referenced by ForceGrowthInitialize().

bool VegElement::m\_forced\_phase\_shift

protected

Referenced by ReduceVeg\_Extended(), SetGrowthPhase(), and VegElement().

double VegElement::m\_green\_biomass

protected

Referenced by GetGreenBiomass(), and RecalculateBugsNStuff().

FILE\* VegElement::m\_ifile

protected

Referenced by ReadBugPercentageFile().

double VegElement::m\_insect\_pop

protected

Referenced by Field::DoDevelopment(), Hedges::DoDevelopment(), HedgeBank::DoDevelopment(), BeetleBank::DoDevelopment(), Scrub::DoDevelopment(), UnsprayedFieldMargin::DoDevelopment(), ForestElement::DoDevelopment(), GetInsectPop(), Insecticide(), InsectMortality(), RecalculateBugsNStuff(), SetCropDataAll(), SetInsectPop(), and VegElement().

double VegElement::m\_LAgreen

protected

Referenced by RoadsideVerge::Cutting(), Orchard::Cutting(), OrchardGrass::Cutting(), DoDevelopment(), Field::DoDevelopment(), ForceGrowthDevelopment(), ForceGrowthInitialize(), GetLAGreen(), RecalculateBugsNStuff(), ReduceVeg(), ReduceVeg\_Extended(), SetCropData(), SetCropDataAll(), SetGrowthPhase(), SetVegHeight(), VegElement(), and ZeroVeg().

double VegElement::m\_LAtotal

protected

Referenced by RoadsideVerge::Cutting(), Orchard::Cutting(), OrchardGrass::Cutting(), DoDevelopment(), Field::DoDevelopment(), UnsprayedFieldMargin::DoDevelopment(), ForceGrowthDevelopment(), ForceGrowthInitialize(), GetLATotal(), RecalculateBugsNStuff(), ReduceVeg(), ReduceVeg\_Extended(), SetCropData(), SetCropDataAll(), SetGrowthPhase(), SetVegHeight(), StoreLAItotal(), VegElement(), and ZeroVeg().

double VegElement::m\_newgrowthsum

protected

Referenced by RecalculateBugsNStuff(), and VegElement().

int VegElement::m\_newoldgrowthindex

protected

Referenced by RecalculateBugsNStuff(), and VegElement().

int VegElement::m\_nutrient\_status

protected

Referenced by VegElement().

double VegElement::m\_oldLAtotal

protected

Referenced by RecalculateBugsNStuff(), and StoreLAItotal().

double VegElement::m\_oldnewgrowth[32]

protected

Referenced by RecalculateBugsNStuff(), and VegElement().

double VegElement::m\_veg\_biomass

protected

Referenced by Field::DoDevelopment(), UnsprayedFieldMargin::DoDevelopment(), GetVegBiomass(), RecalculateBugsNStuff(), ReduceVeg(), ReduceVeg\_Extended(), SetCropDataAll(), VegElement(), and ZeroVeg().

double VegElement::m\_veg\_cover

protected

Referenced by Field::DoDevelopment(), GetVegCover(), RecalculateBugsNStuff(), SetCropData(), SetCropDataAll(), VegElement(), and ZeroVeg().

int VegElement::m\_veg\_density

protected

Referenced by Field::DoDevelopment(), UnsprayedFieldMargin::DoDevelopment(), GetVegDensity(), RecalculateBugsNStuff(), and SetCropDataAll().

double VegElement::m\_veg\_height

protected

Referenced by RoadsideVerge::Cutting(), Orchard::Cutting(), OrchardGrass::Cutting(), DoDevelopment(), Field::DoDevelopment(), UnsprayedFieldMargin::DoDevelopment(), ForceGrowthDevelopment(), ForceGrowthInitialize(), GetVegHeight(), RecalculateBugsNStuff(), ReduceVeg(), ReduceVeg\_Extended(), SetCropData(), SetCropDataAll(), SetGrowthPhase(), SetVegHeight(), VegElement(), and ZeroVeg().

bool VegElement::m\_veg\_patchy

protected

Referenced by Field::DoDevelopment(), GetVegPatchy(), GreenElement::GreenElement(), Heath::Heath(), NaturalGrass::NaturalGrass(), PermanentSetaside::PermanentSetaside(), PermPasture::PermPasture(), PermPastureLowYield::PermPastureLowYield(), PermPastureTussocky::PermPastureTussocky(), RoadsideVerge::RoadsideVerge(), SetCropDataAll(), SetVegPatchy(), UnsprayedFieldMargin::UnsprayedFieldMargin(), and YoungForest::YoungForest().

int VegElement::m\_veg\_phase

protected

Referenced by DoDevelopment(), UnsprayedFieldMargin::DoDevelopment(), and SetGrowthPhase().

TTypesOfVegetation VegElement::m\_vege\_type

protected

Referenced by BeetleBank::BeetleBank(), Field::DoDevelopment(), UnsprayedFieldMargin::DoDevelopment(), FieldBoundary::FieldBoundary(), ForceGrowthSpringTest(), GetVegType(), Heath::Heath(), HedgeBank::HedgeBank(), Hedges::Hedges(), Marsh::Marsh(), NaturalGrass::NaturalGrass(), Orchard::Orchard(), OrchardBand::OrchardBand(), OrchardGrass::OrchardGrass(), PermanentSetaside::PermanentSetaside(), RecalculateBugsNStuff(), RoadsideVerge::RoadsideVerge(), SetCropData(), SetCropDataAll(), SetGrowthPhase(), SetVegType(), UnsprayedFieldMargin::UnsprayedFieldMargin(), VegElement(), and YoungForest::YoungForest().

double VegElement::m\_weed\_biomass

protected

Referenced by DoDevelopment(), Field::DoDevelopment(), UnsprayedFieldMargin::DoDevelopment(), ForceGrowthInitialize(), GetWeedBiomass(), RecalculateBugsNStuff(), ReduceVeg(), ReduceVeg\_Extended(), ReduceWeedBiomass(), SetCropDataAll(), SetGrowthPhase(), SetVegHeight(), VegElement(), and ZeroVeg().

int VegElement::m\_weed\_curve\_num

protected

Referenced by DoDevelopment(), UnsprayedFieldMargin::DoDevelopment(), ForceGrowthInitialize(), SetVegType(), and VegElement().

---

The documentation for this class was generated from the following files:

- elements.h
- elements.cpp


- VegElement
- Generated on Thu Jan 10 2013 13:15:38 for ALMaSS Skylark ODdox by
   1.8.1.1
